# Distinct Responsiveness of Tumor-Associated Macrophages to Immunotherapy of Tumors with Different Mechanisms of Major Histocompatibility Complex Class I Downregulation

**DOI:** 10.3390/cancers13123057

**Published:** 2021-06-19

**Authors:** Adrianna Piatakova, Ingrid Polakova, Jana Smahelova, Shweta Dilip Johari, Jaroslav Nunvar, Michal Smahel

**Affiliations:** Department of Genetics and Microbiology, Faculty of Science, Charles University, BIOCEV, 252 50 Vestec, Czech Republic; adrianna.grzelak@natur.cuni.cz (A.P.); ingrid.polakova@natur.cuni.cz (I.P.); smahelovaj@natur.cuni.cz (J.S.); joharis@natur.cuni.cz (S.D.J.); jaroslav.nunvar@natur.cuni.cz (J.N.)

**Keywords:** repolarization, macrophages, tumor, immunotherapy, major histocompatibility complex, colony-stimulating factor-1

## Abstract

**Simple Summary:**

Tumor-associated macrophages (TAMs) are one of the major cell subpopulations in the tumor microenvironment (TME) where they can either be pro-tumorigenic or contribute to an anti-tumor immunity. The TME and TAM phenotype were analyzed after combined immuno-therapy (IT) in tumor models characterized by distinct expression of major histocompatibility class I complex (MHC-I) molecules, i.e., tumors induced with TC-1 (MHC-I-proficient), TC-1/A9 (reversibly downregulated), and TC-1/dB2m (irreversibly downregulated) cells. We found out that combined IT highly activated immune reactions in the TME of TC-1 and TC-1/A9 tumors, but the TME of TC-1/dB2m tumors remained almost unchanged. Correspondingly, TAMs from TC-1/A9 tumors were able to destroy tumor cells in vitro, while TAMs isolated from TC-1/dB2m tumors showed profoundly decreased cytotoxicity. Hence, various capabilities of TAMs in tumors with distinct expression of MHC-I molecules should be considered when applying IT, particularly IT focused on TAMs.

**Abstract:**

Tumor-associated macrophages (TAMs) plentifully infiltrate the tumor microenvironment (TME), but their role in anti-tumor immunity is controversial. Depending on the acquired polarization, they can either support tumor growth or participate in the elimination of neoplastic cells. In this study, we analyzed the TME by RNA-seq and flow cytometry and examined TAMs after ex vivo activation. Tumors with normal and either reversibly or irreversibly decreased expression of major histocompatibility complex class I (MHC-I) molecules were induced with TC-1, TC-1/A9, and TC-1/dB2m cells, respectively. We found that combined immunotherapy (IT), composed of DNA immunization and the CpG oligodeoxynucleotide (ODN) ODN1826, evoked immune reactions in the TME of TC-1- and TC-1/A9-induced tumors, while the TME of TC-1/dB2m tumors was mostly immunologically unresponsive. TAMs infiltrated both tumor types with MHC-I downregulation, but only TAMs from TC-1/A9 tumors acquired the M1 phenotype upon IT and were cytotoxic in in vitro assay. The anti-tumor effect of combined IT was markedly enhanced by a blockade of the colony-stimulating factor-1 receptor (CSF-1R), but only against TC-1/A9 tumors. Overall, TAMs from tumors with irreversible MHC-I downregulation were resistant to the stimulation of cytotoxic activity. These data suggest the dissimilarity of TAMs from different tumor types, which should be considered when utilizing TAMs in cancer IT.

## 1. Introduction

The majority of the immunotherapeutic approaches for cancer treatment are based on the restoration of cluster of differentiation (CD) 8^+^ cytotoxic T lymphocyte (CTL) activity, which ensures lasting anti-tumor immunity. However, the downregulation of the major histocompatibility complex class I (MHC-I) surface expression, which results in the reduced presentation of antigenic peptides on the tumor cell surface and thus compromises the efficacy of CTLs in tumor cell killing, has been reported in 60–90% of various human tumors, suggesting the crucial role of MHC-I molecules as a prognostic marker and a predictive parameter for efficient immunotherapy (IT) [1,2,3].

Since the number of tumor-infiltrating lymphocytes and macrophages correlated with the number of MHC-I-proficient tumor cells in different tumor types [4,5,6] and structure changes were found in MHC-I negative tumors [6], Garrido et al. hypothesized that the immunoselection of MHC-I-deficient tumor cells leads to tumor encapsulation and peritumoral localization of immune cells [7]. This tumor reorganization that prevents a direct contact of CTLs with tumor cells is associated with a Th2-type immune response and a corresponding macrophage polarization. Unless the interaction between MHC-I molecules presenting epitopes on tumor cells and CTLs can be restored, alternative immunotherapeutic strategies should be considered.

Additionally, emerging data have indicated that novel immunotherapeutic strategies should be based on the coordinated activation of innate and adaptive immunity, and the role of innate immunity in the anti-tumor response should not be underestimated [8,9,10,11]. Moreover, a better understanding of tumor complexity has shown that CTL efficacy is impaired by the immunosuppressive character of the tumor microenvironment (TME) [12]. In this context, IT involving cells of innate immunity, such as macrophages, which abundantly infiltrate the TME and show a high capacity to eliminate neoplastic cells when adequately stimulated, appears as an attractive and complementary strategy [13], especially in the treatment of weakly immunogenic tumors [14,15]. These macrophages are classified as M1 or classically activated macrophages and can exert an anti-tumor effect through the production of high levels of proinflammatory cytokines, such as interleukin (IL)-12 and tumor necrosis factor (TNF)-α, as well as radical oxygen and nitrogen species, such as nitric oxide (NO), which is produced by the enzyme inducible nitric oxide synthase (iNOS). Additionally, M1 macrophages express higher levels of MHC-II molecules [16]. In contrast, M2 or alternatively activated macrophages are characterized by the production of IL-10 and transforming growth factor (TGF)-β and increased enzymatic activity of arginase 1 and indoleamine-2,3-dioxygenase 1 (IDO1). The majority of tumor-associated macrophages (TAMs) are of the M2 phenotype, which is associated with the suppression of anti-tumor immunity and the support of tumor growth [12]. Hence, the repolarization of TAMs to the M1 phenotype has recently attracted increased attention [13].

In fact, the TMEs of solid tumors are populated by highly heterogenous TAMs with various functions [16], which can undergo a switch between pro- and anti-tumor phenotypes in different stages of tumor development and progression [17]. Additionally, contrasting data show that macrophages can exert in vitro cytotoxicity against various tumor cells through distinct mediators, such as NO and TNF-α [18]—these being hallmarks of M1 macrophages—but also arginase [19], which is a crucial characteristic of M2 macrophages. Moreover, the co-existence of TAMs and CD8^+^ T cells in the TME and their anti-tumor effect are complex. On one hand, TAMs can inhibit migration of CD8^+^ T cells to the tumor, resulting in formation of an immune-excluded phenotype [20]; on the other hand, the cooperation of CTLs and macrophages is essential for tumor elimination after immunotherapy [21]. Therefore, the complexity and dynamics of the TAM phenotype skewing in the TME raise questions about the time point at which TAMs should be immunotherapeutically targeted and whether TAM elimination from the TME is always beneficial in cancer treatment.

One of the most promising therapeutic targets for the repolarization of TAMs for cancer treatment is a blockade of the colony-stimulating factor (CSF)-1/colony-stimulating factor-1 receptor (CSF-1R) pathway. CSF-1 was initially described as a cytokine involved in macrophage differentiation and proliferation [22]. Recently, it has been demonstrated that, by inhibiting CSF-1R, TAMs can reprogram from the M2 to the M1 phenotype in the TME, work in concert with T cells, and participate in tumor elimination [23,24,25]. Simultaneously, some TAM subpopulations can be depleted upon the CSF1-R blockade [26].

In this study, we investigated the TME and the polarization of TAMs in tumors with normal (TC-1) and either reversibly (TC-1/A9) or irreversibly (TC-1/dB2m) decreased expression of MHC-I molecules. The characteristics of TAMs were also examined through in vitro assays. Combined IT, composed of DNA immunization and intraperitoneal (i.p.) injection of a CpG oligodeoxynucleotide (ODN), ODN1826, resulted in an inflammatory TME in TC-1 and TC-1/A9 tumors; however, the TME in TC-1/dB2m tumors remained almost unaffected. Similarly, TAMs could only be activated in TC-1 and TC-1/A9 tumors. Moreover, while TAMs isolated from TC-1/A9 tumors were stimulated in vitro, and both NO and TNF-α contributed to their cytotoxic effect against tumor cells, TC-1/dB2m TAMs showed a decreased capability for polarization and cytotoxicity. Taken together, these data demonstrate differences in responsiveness to the activation of TAMs from tumors with various MHC-I expressions and, hence, highlight the importance of their potentially different contributions to the anti-tumor immunity induced by IT.

## 2. Materials and Methods

### 2.1. Mice

Seven- to eight-week-old and seven- to ten-week-old female C57BL/6NCrl mice (Charles River, Sulzfeld, Germany) were used for the immunization and ex vivo experiments, respectively. The animal experiments were conducted at the Animal Facility of the Czech Center of Phenogenomics (BIOCEV, Vestec, Czech Republic).

### 2.2. Tumor Cell Lines

Three mouse tumor cell lines were used in the experiments. TC-1 cells were developed by the transformation of primary C57BL/6 mouse lung cells with human papillomavirus type 16 (HPV16) *E6/E7* oncogenes and activated *H-ras* [27]. The TC-1/A9 clone was derived from a tumor induced with TC-1 cells in a mouse that had been preimmunized against the E7 antigen. The TC-1/A9 clone is characterized by a reversible MHC-I reduction that can be upregulated both in vivo and in vitro with interferon (IFN)-γ treatment [28]. The TC-1/dB2m clone was developed in vitro by deactivating the *B2m* gene in TC-1 cells with the CRISPR/Cas9 system and is characterized by irreversible surface MHC-I downregulation [29]. All cells were cultured in high-glucose Dulbecco’s Modified Eagle’s Medium (DMEM; Sigma-Aldrich, St. Louis, MO, USA, D6429), supplemented with 10% fetal bovine serum (FBS; Biosera, Nuaille, France, FB-1090), 100 U/mL penicillin, and 100 μg/mL streptomycin (DMEM-K).

### 2.3. Plasmid

The pBSC/PADRE.E7GGG [30] plasmid was used in immunization experiments. The *PADRE.E7GGG* fusion gene encompasses the HPV16 *E7* oncogene, containing three point mutations in the sequence coding for the Rb binding site (E7GGG) [31] and the sequence encoding Pan DR helper epitope (PADRE), designed in silico [32].

### 2.4. Combined IT

C57BL/6NCrl mice were injected with 3 × 10^4^ TC-1, 3 × 10^4^ TC-1/A9, or 3 × 10^5^ TC-1/dB2m cells suspended in 0.15 mL phosphate-buffered saline (PBS). The cells were inoculated by subcutaneous (s.c.) injection into the backs of animals under anesthesia (day 0). Next, mice were immunized with the pBSC/PADRE.E7GGG plasmid using a gene gun (Bio-Rad, Hercules, CA, USA), delivering 2 μg of plasmid DNA in two shots on days 10, 13, and 17 after the inoculation of TC-1 or TC-1/dB2m cells, and on days 3, 6, and 10 after TC-1/A9 administration. DNA immunization was performed at a discharge pressure of 400 psi into shaven abdomen skin. Following this, a dose of 50 μg CpG ODN1826 (class B; TCCATGACGTTCCTGACGTT; Generi Biotech, Hradec Kralove, Czech Republic) was i.p. injected in 200 μL PBS into the immunized mice. The mice injected with TC-1 and TC-1/A9 cells received 3 doses of ODN1826 on days 17, 20, and 24, and days 10, 13 and 17, respectively. Mice with TC-1/dB2m tumors received 7 doses of ODN1826, beginning from day 17 with 3–4-day intervals. For the analysis of the TME and TAMs, the treated tumors were harvested 2 and 9 days after the completion of combined IT, while control naïve tumors were collected on days 17, 12, and 33 for TC-1, TC-1/A9, and TC-1/dB2m tumors, respectively.

In some experiments, 300 μg anti-TGF-β (clone 1D11.16.8), anti-CSF-1R (clone AFS98), or anti-IL-10 (clone JES5-2A5) monoclonal antibodies (Bio X Cell, West Lebanon, NH, USA), diluted in 200 μL PBS, were injected i.p. into mice. To inhibit arginase activity, 1 mg N-ω-Hydroxy-L-norarginine acetate salt (nor-NOHA; BACHEM, Bubendorf, Switzerland, 6370), dissolved in 200 μL PBS, was also inoculated i.p. The neutralization and inhibition of enzyme activity were performed between days 7 and 28 or 14 and 39 after TC-1/A9 or TC-1/dB2m tumor cell inoculation, respectively, with 3–4-day intervals. In total, the mice with TC-1/A9 tumors received 7 doses of the antibodies or inhibitor, while 8 doses of these compounds were administered to the animals carrying TC-1/dB2m-induced tumors, applied the day after injection of ODN1826.

For the in vivo experiment that compared the efficacy of anti-CSF-1R therapy in TC-1/A9-induced tumors, immunized mice were injected with the ODN1826 adjuvant on days 10, 13, and 17 with a one-week delay with respect to the DNA immunization; otherwise, they were injected 7 times with 4 additional doses on days 21, 24, 28, and 31. Anti-CSF-1R was applied 7 times on days 7, 11, 14, 18, 21, 25, and 28. In a group receiving a combination of ODN1826 and anti-CSF-1R, ODN1826 was administered 3 times on days 10, 13, and 17, while anti-CSF-1R was applied 7 times on days 7, 11, 14, 18, 21, 25, and 28 after tumor cell inoculation.

Tumor progression was controlled three times per week using a caliper, and the tumor volume was calculated according to the formula $\frac{\pi }{6}\left(a\times b\times c\right)$, where *a*, *b*, and *c* are the length, width, and height of a tumor.

### 2.5. NGS Library Preparation and Sequencing

Total RNA for next-generation sequencing (NGS) was isolated from mouse tumor samples (3 per group) with the NucleoSpin RNA kit (Macherey Nagel, Düren, Germany), according to the manufacturer’s protocol, as described previously [15]. The integrity of the RNA was determined by the Experion RNA StdSens assay (Bio-Rad Laboratories), with an RNA integrity number of higher than 9.3 for all samples. NGS libraries were prepared from 500 ng total RNA using the QuantSeq 3′mRNA-Seq Library Prep Kit (FWD) for Illumina with single indexing according to the manufacturer´s protocol (Lexogen, Vienna, Austria). The quality of the libraries was evaluated by the High Sensitivity DNA assay on a 2100 Expert Bioanalyzer (Agilent Technologies, Santa Clara, CA, USA). Libraries were quantified by the Qubit Fluorometer (Thermo Fisher Scientific, Waltham, MA, USA) and subsequently pooled at equimolar ratios. Sequencing was performed on the NextSeq 500 System (Illumina, San Diego, CA, USA) with 75 bp reads at the Core Facility for Genomics and Bioinformatics (Institute of Molecular Genetics, Prague, Czech Republic).

### 2.6. RNA-Seq Data Analysis

As recommended by the library preparation kit manufacturer, sequencing reads were trimmed of adapters, the first 12 nucleotides (5′end), poly(A), and low-quality regions (3′end) using BBDuk (https://sourceforge.net/projects/bbmap/, accessed on 1 February 2021). The trimmed reads were mapped onto the mouse reference genome assembly, GRCm38.p6 (GCF_000001635.26), using the Geneious RNA mapper (Geneious Prime 2020; https://www.geneious.com, accessed on 1 February 2021). The complete analysis of differential expression (DE) between triplicate tumor samples (read count determination, expression normalization, DE calculation, calculation of statistical significance) was carried out using the DESeq2 package [33]. Genes exhibiting a fold change in expression (FC) of ≥2.0 and a statistical support of padj (*p*-value adjusted for multiple testing) of ≤0.1 were considered differentially expressed and evaluated by an enrichment analysis using Enrichr [34,35]. Immune-related genes expressed in examined tumors (*n* = 173; Table S1) were selected and categorized based on previous studies [36,37,38]. Clustering of tumor samples based on the similarity of the expression of these genes was analyzed using principal component analysis (PCA) and hierarchical clustering with ClustVis [39], using default parameters. Gene sets associated with macrophages (*n* = 308) were selected from all of the 9 major collections of the Molecular Signature Database v7.2, available online (https://www.gsea-msigdb.org/gsea/msigdb/index.jsp, accessed on 2 February 2021), and analyzed by single-sample gene set enrichment analysis (ssGSEA) with default settings using the GenePattern public server (https://www.genepattern.org/, accessed on 2 February 2021) and, subsequently, ClustVis.

### 2.7. Flow Cytometry

For flow cytometry analysis, the preparation of a single cell suspension from harvested tumors was carried out as described previously [40]. Two panels of fluorescent-labeled antibodies (Table 1) were used to characterize the main myeloid cell populations: the first panel in the TME characterization experiment and the second panel after combined IT, enhanced by anti-CSF-1R administration. The gating strategies are presented in Figures S1 and S2. Viability staining was performed with Fixable Viability Dye eFluor506 (Thermo Fisher Scientific, Waltham, MA, USA), diluted in PBS with subsequent surface staining. For intracellular staining, the cells were first fixed with IC Fixation Buffer (eBioscience, San Diego, CA, USA, 00-8222-49) followed by permeabilization and washing steps with permeabilization buffer (eBioscience, San Diego, CA, USA, 00-8333-56). The stained cells were measured on the Cytoflex LX (Beckman Coulter, Indianapolis, IN, USA) flow cytometer and analyzed using FlowJo software v10.7.1 (BD Biosciences).

### 2.8. Isolation of TAMs

For experiments performed on TAMs in vitro, 3 × 10^5^ TC-1/A9 or 1 × 10^6^ TC-1/dB2m cells were inoculated s.c. into mice. Non-necrotic TC-1/A9- and TC-1/dB2m-induced tumors were excised on days 13–14 or 35–40, respectively. The harvested tumors were digested with 1 mg/mL collagenase NB 8 (SERVA, Heidelberg, Germany, 17456) and 100 μg/mL DNase I (SERVA, 8535) in Roswell Park Memorial Institute (RPMI) 1640 medium (without FBS; Sigma-Aldrich, Merck, KGaK, R8758) at 37 °C using the gentleMACS Octo Dissociator (Miltenyi Biotec, Bergisch Gladbach, Germany), and the cell suspension was filtered through a 70 μm mesh (Miltenyi Biotec). After the removal of erythrocytes with ACK buffer (0.15 M NH_4_Cl, 10 mM KHCO_3_, 0.5 M EDTA, pH 7.2–7.4), F4/80^+^ cells were enriched using anti-F4/80^+^ antibody-conjugated magnetic beads (Miltenyi Biotec, 130-110-443) and the autoMACS Pro Separator (Miltenyi Biotec), according to the manufacturer’s instructions. The collected F4/80^+^ cells were used for further experiments. The cells were cultured in DMEM F12 (Biosera, Nuaille, France, LM-D1222) with 10% FBS and antibiotics, as described above (DMEM F12/10).

### 2.9. In Vitro Stimulations of TAMs

For co-cultures, 75 × 10^3^ of viable TAMs were plated on a 96-well plate in 100 μL DMEM F12/10 per well and incubated overnight at 37 °C in 5% CO_2_. Then, the cells were washed with warm PBS and overlaid with 7.5 × 10^3^ tumor cells in 100 μL DMEM-K. Control cells, i.e., TAMs without tumor cells and tumor cells alone, were also incubated in DMEM-K. As soon as the tumor cells adhered, the cells (TAMs, tumor cells, and the co-cultures) were washed with warm PBS, followed by the application of stimulatory compounds for 44 h. For enzyme-linked immunosorbent assay (ELISA), 3 × 10^5^ TAMs were seeded in a 24-well plate and stimulated, as described below.

The cells were stimulated with the following reagents or their combinations: 5 μg/mL ODN1826, 200 U/mL IFN-γ (PeproTech, Rocky Hill, NJ, USA, 315-05), 10 ng/mL lipopolysaccharide (LPS; Sigma-Aldrich, L4391), 25 ng/mL IL-4 (PeproTech, 214-14). The M1 phenotype was induced with a combination of IFN-γ + LPS, and stimulation with IL-4 was used for M2 polarization. Unstimulated cells were used as a negative control. To evaluate the role of NO and TNF-α cytotoxicity against tumor cells, the iNOS inhibitor Nω-Nitro-L-arginine methyl ester hydrochloride (L-NAME; Sigma-Aldrich, N5751) and anti-TNF-α (clone MP6-XT3, eBioscience, San Diego, CA, USA), or the respective isotype control for rat IgG1 kappa (clone eBRG1, eBioscience), were added to the stimulations. At the indicated time, the supernatants were collected and assessed for nitrite concentration to determine iNOS activity, while the cells that had been washed with warm PBS were used for a 3-(4,5-dimethylthiazol-2-yl)2,5-diphenyl tetrazolium bromide (MTT) assay. During the enzyme shift investigation experiments, the cell culture supernatants were collected and assessed for nitrite concentration, while the cell lysates were used for an arginase microplate assay.

### 2.10. MTT Cytotoxicity Assay

The test was adopted from [41] with some modifications. The MTT (Duchefa Biochemie, Haarlem, the Netherlands, M1415) stock, with a 5 mg/mL concentration, was diluted in DMEM-K to achieve 0.5 mg/mL and mixed well. A volume of 110 μL was gently added to the cells in each of the 96 wells and the plate was incubated at 37 °C for 3.5 h. Then, 100 μL of 10% SDS in 0.04 N HCl solution was added to the wells, and the formazan crystals were dissolved overnight at 37 °C. The absorbance was measured at 570 nm with a background of 690 nm using a microplate reader (Tecan, Männedorf, Switzerland). The percentage of cytotoxicity was calculated according to the following formula:

$$Cytotoxicity\;\left[\%\right]=100-\left(\frac{\left[OD_{co-culture}\right]-\left[OD_{TAMs}\right]}{\left[OD_{tumor\;cells}\right]}\right)\times 100$$

### 2.11. NO Measurement

The NO concentration was measured in the form of NO_2_^−^, which is easily detected with Griess reagent, composed of 0.2% naphthylethylenediamine dihydrochloride (Sigma-Aldrich, 222488) and 2% sulphanilamide (Sigma-Aldrich, S9251) in 5% phosphoric acid solution, as previously described [15]. Briefly, equal volumes of the cell culture supernatant and Griess reagent were mixed, and the absorbance was measured at 540 nm using a microplate reader (Tecan). The nitrite concentration was determined by means of the sodium nitrite standard curve (0–100 μM; Sigma-Aldrich, 31443).

### 2.12. ELISA

The concentrations of cytokines IL-12p70, IL-10, and TNF-α were measured by Invitrogen ELISA Kits (Thermo Fisher Scientific; 88-7121-22, 88-7105-22, and 88-7324-22, respectively) in the supernatants from in vitro stimulations of TAMs. Prior to the measurements, the supernatants were centrifuged for 5 min at 350× *g*.

### 2.13. Arginase Microplate Assay

Arginase activity was measured in cell lysates with the microplate method, where urea was detected by colorimetry, as previously described [42]. Absorbance was measured at 540 nm using a microplate reader, and the urea concentration was determined by means of the standard curve between 0 and 640 μg/mL.

### 2.14. Statistical Analysis

Intergroup comparisons of ex vivo flow cytometry and arginase assay were performed by one-way analysis of variance (ANOVA) with Tukey’s multiple comparisons, while the results from the ELISA were analyzed by one-way ANOVA with Dunnett’s multiple comparisons. Student’s two-tailed *t*-test was used to determine the influence of NO or TNF-α in the experimental and control groups for one stimulation. Tumor growth was evaluated using two-way ANOVA with Dunnett’s multiple comparison test. A difference between groups was considered statistically significant if *p* < 0.05. Calculations were performed using the GraphPad Prism 8 software (GraphPad Software, San Diego, CA, USA).

## 3. Results

### 3.1. Microenvironments of TC-1/A9 and TC-1/dB2m Tumors Differ in Immune Reactions

To study the TME of tumors with distinct types of MHC-I expression, we performed RNA-seq analysis of samples isolated from naïve tumors and tumors treated by combined IT, consisting of DNA vaccination and the i.p. administration of the CpG ODN1826 adjuvant. Due to the variability in immunogenicity of the used cell lines and the growth of induced tumors, different experimental conditions were used for each cell line (see Materials and Methods). Treated tumors were collected either 2 days after IT termination when tumors induced with TC-1/A9 cells were usually partially regressed, or 9 days after IT when the growth of TC-1/A9-induced tumors was restored [15].

**Figure 1:**
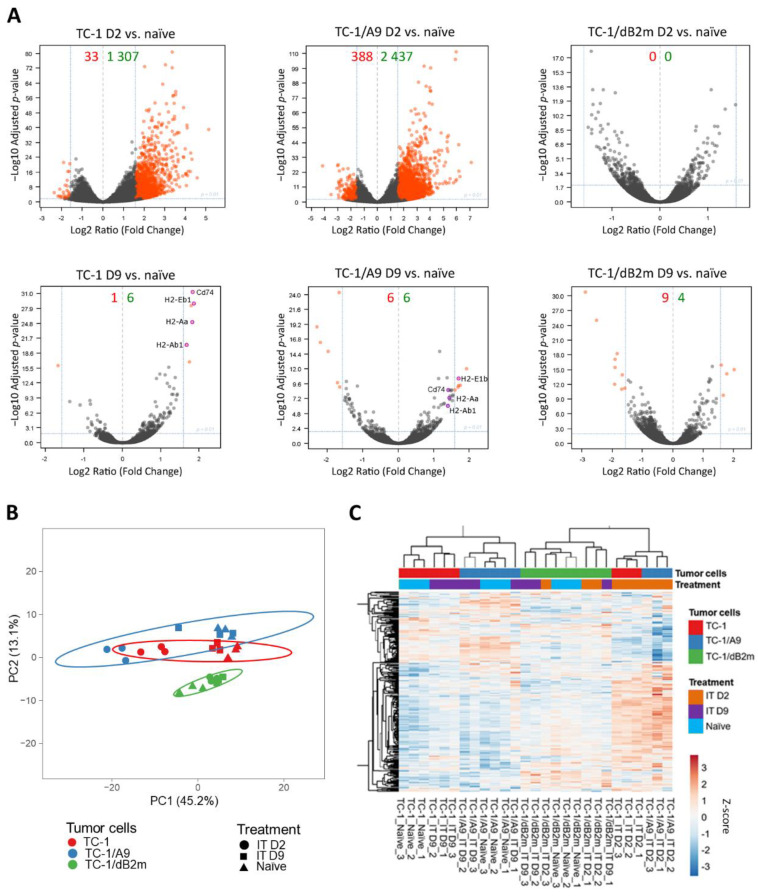
RNA-seq analysis of the TME. RNA was isolated from naïve tumors and tumors 2 (D2) or 9 days (D9) after IT termination. (**A**) Differential expression of all detected genes. Orange dots indicate genes with at least 3-fold increased or decreased expression and padj ≤0.01. Numbers of these genes are indicated in green and red colors, respectively. (**B**) PCA of the expression of immune-related genes. (**C**) Cluster analysis of gene sets associated with macrophages.

The evaluation of differentially expressed genes showed that the TME of TC-1- and TC-1/A9-induced tumors responded similarly to IT, but their response differed from TC-1/dB2m tumors (Figure 1A). Two days after treatment, the TME was greatly altered in TC-1 and TC-1/A9 tumors in comparison to the TME in naïve tumors. This effect was more prominent in TC-1/A9-induced tumors. The spectrum of genes which exhibited increased expression indicated an inflammatory reaction and the activation of both innate and adaptive immunity. However, 9 days after IT, the TME in treated tumors more closely resembled that of naïve tumors. In TC-1/dB2m tumors, the TME was only slightly altered by IT without consideration for the interval of tumor collection after treatment.

Enrichment analysis of differentially expressed genes showed that the main difference between TC-1 and TC-1/A9 tumors 2 days after IT was an approximately 6-fold greater expression of *S100a8*, *S100a9*, *Camp*, *Lcn2*, and *Ltf* genes associated with neutrophil activation and degranulation in TC-1/A9 tumors (Figure S3A). In both tumor types, MHC-II genes mostly remained upregulated 9 days after IT (Figure 1A). For TC-1/dB2m tumors, the number of differentially expressed genes was low, and therefore no enrichment was recorded after treatment. From immune-related genes, the increased expression of some chemokines and their receptors (*Cxcl12*, *Cx3cl1*, *Ccr1*, *Ccr2*, *Ccr7*, *Ccr9*, and *Cx3cr1*) was found in naïve TC-1/dB2m tumors in comparison with TC-1 tumors (Figure S3B).

Further evaluation of the RNA-seq data of immune-related genes (Table S1) by PCA (Figure 1B) and the cluster analysis (Figure S4) confirmed that immune reactions were increased in TC-1 and TC-1/A9 tumors 2 days after IT and were minimally affected in TC-1/dB2m tumors. Next, we found a cluster of genes with higher expression in TC-1/A9- than in TC-1-induced tumors 2 days after IT (Figure S4). The cluster analysis of gene sets, associated with macrophages and evaluated by ssGSEA (Figure 1C), resulted in a pattern similar to that of immune-related genes. This outcome suggests a slight difference in macrophages between naïve TC-1- and TC-1/A9-induced tumors, and a marked alteration in these cells 2 days after IT. In TC-1/dB2m tumors, the gene sets associated with macrophages exhibited a distinct expression pattern that did not substantially change after IT.

In summary, in TC-1/A9 tumors with a reversible reduction in MHC-I molecules, similar to TC-1 tumors, IT effectively induced immune reactions in the TME, while TC-1/dB2m tumors with irreversible MHC-I downregulation were only slightly affected. Correspondingly, the expression patterns of the macrophage-associated gene sets in TC-1 and TC-1/A9 tumors were similar, but they differed in TC-1/dB2m-induced tumors.

### 3.2. TAMs from Tumors with Distinct MHC-I Expression Differ in Their Phenotype and Response to IT

In parallel to the RNA-seq analysis of the TME in tumors with distinct MHC-I expression, we characterized TAM phenotypes at different stages of tumor progression using flow cytometry. Tumors were subjected to the same immunotherapeutic treatment schedule and harvested on the same days, as described above.

**Figure 2:**
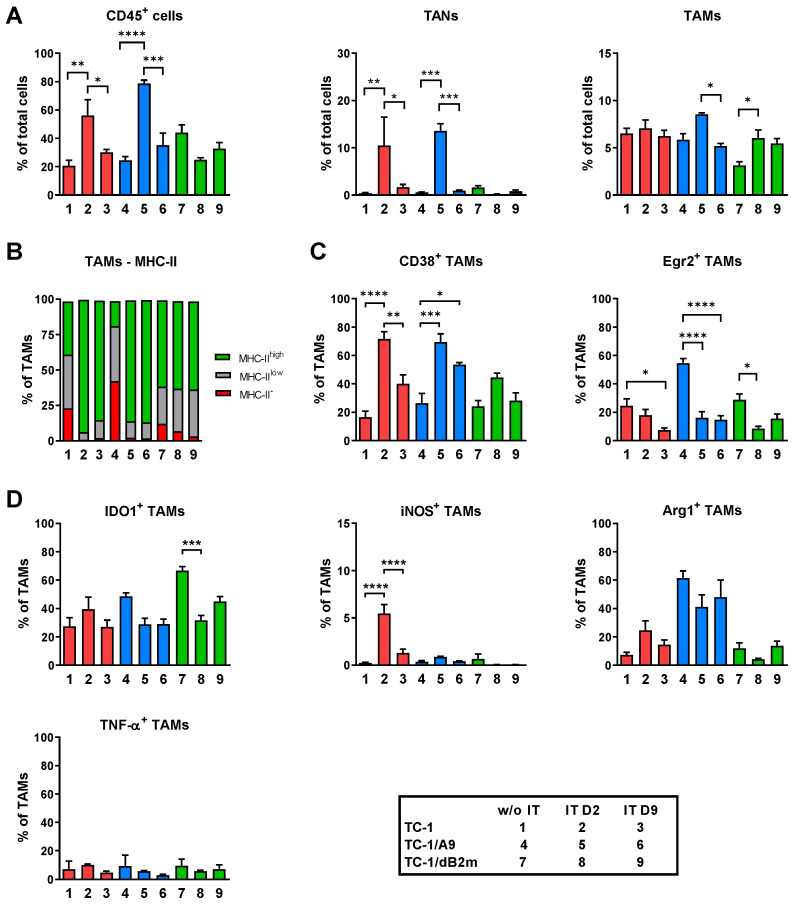
Ex vivo characterization of TAMs. Mice (*n* = 4) were injected with tumor cells and subsequently treated with combined IT, consisting of immunization by a gene gun and i.p. injection of the ODN1826 adjuvant, as described in Material and Methods. On days 2 (D2) and 9 (D9) after completion of treatment, cells were isolated from tumors, and myeloid cells were analyzed by flow cytometry. Non-treated tumors were used as a control. (**A**) Infiltration of tumors with immune cells (CD45^+^), TANs (CD11b^+^Ly6G^high^Ly6C^low^), and TAMs (CD11b^+^Ly6G^−^Ly6C^−^F4/80^+^). (**B**) Overview of the mean percentages of TAM subpopulations as determined by MHC-II expression. (**C**) Analysis of M1 and M2 TAM frequencies using CD38 and Erg2 markers, respectively. (**D**) Frequencies of TAMs expressing the amino acid-degrading enzymes iNOS, arginase 1, and IDO1, and the cytokine TNF-α. Bars: ± SEM; * *p* < 0.05, ** *p* < 0.01, *** *p* < 0.001, **** *p* < 0.0001.

In the non-treated mice, the numbers of CD45^+^ cells were comparable for TC-1- and TC-1/A9-induced tumors, constituting about 20% of total live cells (Figure 2A). Two days after the completion of IT, the numbers of these cells significantly increased in both tumor types, and after another week, they dropped down to a level comparable with that of the naïve tumors. Combined IT did not induce significant alterations in the proportion of CD45^+^ cells in TC-1/dB2m-induced tumors throughout the experimental period. Infiltration kinetics of tumors with tumor-associated neutrophils (TANs; CD11b^+^Ly6G^high^Ly6C^low^) reflected that observed for CD45^+^ infiltration. While combined IT increased the TAN infiltration of TC-1- and TC-1/A9-induced tumors, it did not promote TAN migration to the TC-1/dB2m tumors. In contrast to the variable numbers of CD45^+^ cells and TANs, the number of TAMs (CD11b^+^Ly6G^−^Ly6C^−^F4/80^+^) remained unchanged in TC-1-tumors, while the number of TAMs infiltrating TC-1/A9- and TC-1/dB2m-induced tumors was slightly increased after combined IT (Figure 2A). As in our former reports [15,29], we analyzed a TAM polarization state through the expression of MHC-II molecules, which is regarded as an M1 macrophage marker (Figure 2B). We observed the increase in the surface expression of MHC-II molecules only in the treated TC-1- and TC-1/A9-induced tumors, while the majority of TAMs from TC-1/dB2m-induced tumors expressed high levels of MHC-II regardless of the treatment. To better distinguish TAM phenotypes, we applied another classification system in which CD38 and Early growth response protein 2 (Egr2) are used to define M1 and M2 murine macrophages, respectively [43]. As demonstrated in Figure 2C, Egr2^+^ TAMs decreased in all tumor types after combined IT. Simultaneously, the proportion of CD38^+^ TAMs increased, but this increase was only significant in treated TC-1 and TC-1/A9 tumors in this experiment.

Finally, we monitored the functional state of TAMs in tumors and aimed to determine the expression of representative macrophage markers: iNOS and TNF-α for the M1 phenotype, or arginase 1 and IDO1 enzymes for which expression is elevated in M2 macrophages (Figure 2D). After combined IT, the expression of iNOS was only significantly enhanced in TC-1-induced tumors. However, this effect was temporary, as, after one week, its expression markedly decreased. TAMs expressing arginase 1 were found in all three types of tumors, but their number was not significantly affected by combined IT. The expression of IDO1 was comparable in TC-1- and TC-1/A9-formed tumors, while the proportion of IDO1^+^ TAMs had already significantly reduced by 2 days following IT completion in TC-1/dB2m tumors. Finally, the expression of TNF-α in all tumor types was low and uninfluenced by the treatment.

Taken together, these data demonstrate different TAM phenotypes and reactivity to combined IT in tumors with different MHC-I expression levels.

### 3.3. TAMs from Tumors with Irreversible MHC-I Downregulation Were Not Cytotoxic against Tumor Cells

**Figure 3:**
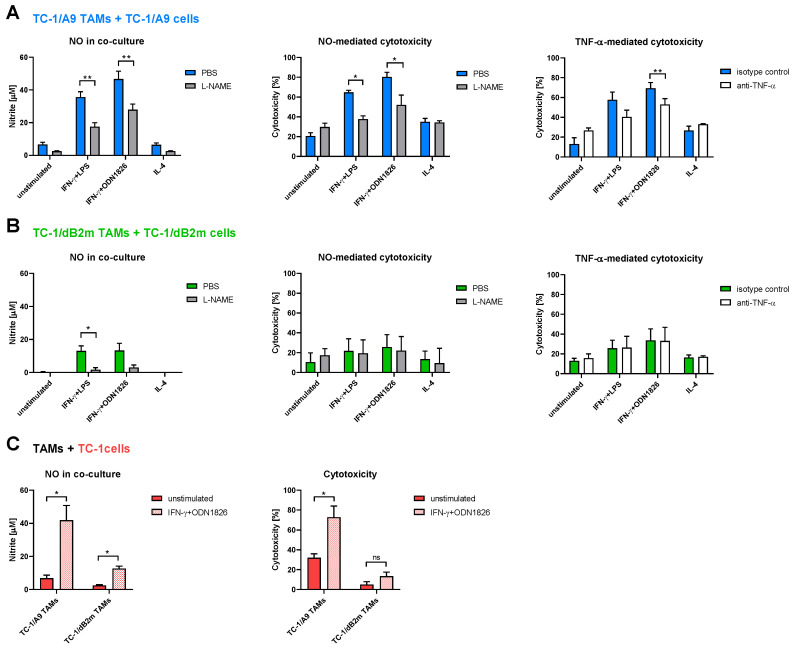
In Vitro cytotoxicity of TAMs against tumor cells. Co-cultures of TAMs with tumor cells were established and stimulated with IL-4 or IFN-γ + TLR agonist for 44 h. The unstimulated cells were used as a negative control. The influence of NO and TNF-α on cytotoxicity was tested with L-NAME and anti-TNF-α, respectively (**A**,**B**). Subsequently, the supernatants from co-cultures were collected and nitrite concentrations were measured with Griess reagent (**A**–**C**; left-hand graphs). Next, the cells were used for the MTT assay (**A**–**C**; middle and right-hand graphs). Co-cultures were set in the following combinations: (**A**) TAMs from TC-1/A9 tumors plus TC-1/A9 cells; (**B**) TAMs from TC-1/dB2m tumors plus TC-1/dB2m cells; (**C**) TAMs from TC-1/A9 tumors or TC-1/dB2m tumors plus TC-1 cells. Columns indicate mean of three independent experiments; bars: ± SEM; ns; non-significant, * *p* < 0.05, ** *p* < 0.01.

Aside from the ex vivo analysis of the TME and TAMs from tumors with distinct MHC-I expression, we set up a panel of in vitro cytotoxicity tests for the evaluation of TAMs’ potential to inhibit the proliferation of tumor cells with MHC-I downregulation. To this end, TAMs isolated from TC-1/A9-induced tumors were co-cultured with TC-1/A9 cells, while TAMs from TC-1/dB2m-induced tumors were mixed with TC-1/dB2m cells. These co-cultures, as well as TAMs and tumor cells alone, were stimulated with IL-4 or an IFN-γ plus a Toll-like receptor (TLR) agonist (either LPS or ODN1826), or they were left unstimulated (Figure 3). Additionally, as NO and TNF-α are well-known cytotoxic mediators generated in vitro by macrophages against various tumor cell lines [18,44], we tested the effect of their neutralization on the ability of TAMs to kill tumor cells. Therefore, an MTT assay was performed on TAMs, cultivated with tumor cells in the presence of L-NAME, the iNOS inhibitor, or the neutralizing antibody against TNF-α. In a co-culture, TAMs from TC-1/A9 tumors that were stimulated with a combination of IFN-γ + TLR agonist (M1-TAMs) produced high amounts of NO, which corresponded to the greater cytotoxic effect against TC-1/A9 cells when compared to the unstimulated or IL-4-stimulated cells (M2-TAMs). Upon the addition of the L-NAME inhibitor, which decreased the production of nitrite in a co-culture, the significant reduction in the tumoristatic effect of TC-1/A9 TAMs against TC-1/A9 cells was observed (Figure 3A). In a co-culture of IFN-γ + TLR ligand-polarized TC-1/dB2m TAMs with TC-1/dB2m cells, low levels of nitrite were observed (Figure 3B). Accordingly, TAMs demonstrated low cytotoxicity against these tumor cells. Reducing the levels of nitrite with the L-NAME compound did not affect cytotoxicity, suggesting that NO did not contribute to the cytotoxic effect of TC-1/dB2m TAMs against TC-1/dB2m cells. Similarly, TNF-α only contributed to the cytotoxic activity of TC-1/A9 TAMs when stimulated with the IFN-γ + TLR agonist (Figure 3A,B; right-hand graphs).

To rule out the possibility of variability in the antiproliferative potential of TAMs against various tumor cells, we evaluated the cytotoxic ability of both types of TAMs against the same target, TC-1 tumor cells (Figure 3C). We stimulated co-cultures with a combination of IFN-γ and ODN1826—as this combination resembled the IT from our in vivo experiments—and compared them to the unstimulated co-cultures with the concomitant quantification of nitrite in cell culture supernatants. While M1-polarized TAMs from TC-1/A9 tumors produced high levels of nitrite, which correlated with the increased cytotoxicity, the level of nitrite in the co-culture of M1-polarized TC-1/dB2m TAMs with TC-1 cells was lower and did not significantly affect the cytotoxicity. Hence, these results demonstrate a different cytotoxic potential of TAMs from TC-1/A9- and TC-1/dB2m-induced tumors, mediated by both NO and TNF-α in TAMs from TC-1/A9 tumors.

### 3.4. In Vitro Inhibition of iNOS Did Not Increase Arginase Activity in TAMs

We have previously demonstrated that TAMs isolated from TC-1/A9 tumors, as adherent CD45^+^ cells, upregulated iNOS and arginase in co-culture with TC-1/A9 cells, depending on the type of stimulation [15]. In this experimental design, we compared the ability of F4/80^+^ TAMs from TC-1/A9 and TC-1/dB2m tumors to induce both enzymes in co-culture with TC-1 tumor cells. Since the interaction of these enzymes is strictly connected—aside from sharing the common substrate, arginase has been reported to be a regulator of iNOS activity [45,46]—we also investigated whether the shift in activities between the enzymes is feasible, which may suggest the possibility of using enzyme modulation for IT. Additionally, both arginase and iNOS utilize arginine for enzymatic conversions, but their reaction products are different, which may further influence the events taking place in the TME and the response to IT [47].

**Figure 4:**
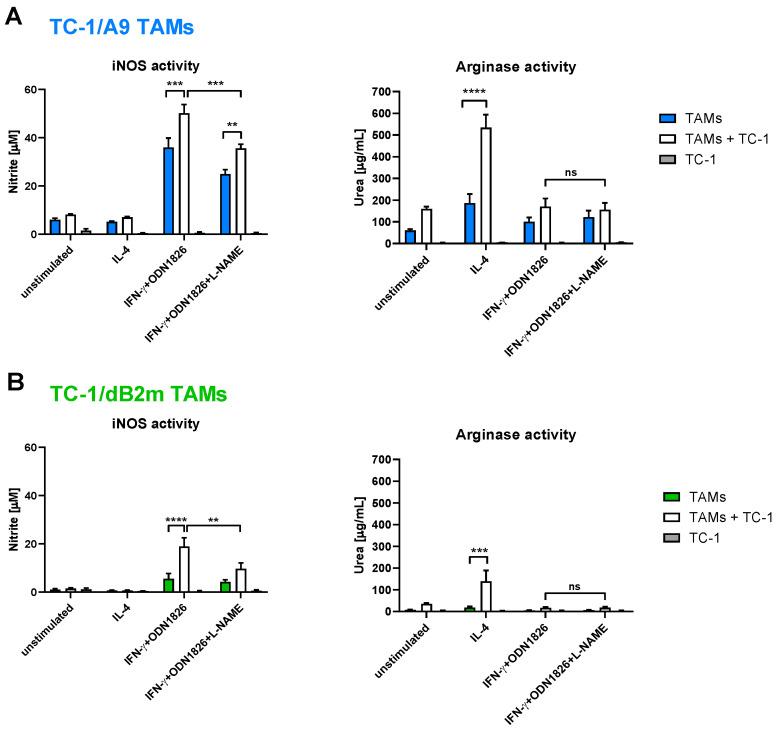
In Vitro modulation of arginine-metabolizing enzymes in co-cultures of TAMs with tumor cells. TAMs, TC-1 cells, and co-cultures of TAMs from TC-1/A9 (**A**) or TC-1/dB2m tumors (**B**) with TC-1 cells were stimulated with IL-4 or IFN-γ plus ODN1826 for 44 h. The activity of iNOS in M1 TAMs was blocked with L-NAME. Subsequently, supernatants from co-cultures were collected, and nitrite concentration and arginase enzymatic activity were measured with Griess reagent (**A**,**B**; left-hand graphs) and by microplate method on cell lysates (**A**,**B**; right-hand graphs), respectively. The unstimulated cells were used as a negative control. Columns indicate mean of three independent experiments; bars: ± SEM; ns; non-significant, ** *p* < 0.01, *** *p* < 0.001, **** *p* < 0.0001.

The co-culture with TC-1 cells significantly increased iNOS activity in both types of TAMs that were stimulated with the combination of IFN-γ and ODN1826 (Figure 4A,B; left-hand graph), while arginase activity was markedly enhanced in IL-4-activated TAMs by co-cultivation with these tumor cells (Figure 4A; right-hand graph). Overall, TAMs from TC-1/dB2m tumors showed lower activities of iNOS and arginase, while TC-1/A9 TAMs could easily upregulate both enzymes.

Considering the enzymatic shift between arginine-degrading enzymes, we blocked the iNOS activity with L-NAME and subsequently tested whether the arginase activity was augmented as a result of the increase in arginine availability. Therefore, we first measured the nitrite concentrations in cell culture media and then lysed the cells for the arginase microplate assay. The application of L-NAME resulted in a decrease in the nitrite concentration in both types of TAMs after IFN-γ plus ODN1826 stimulation (Figure 4). However, no difference was observed in the arginase activity between these two groups. Therefore, blocking iNOS with the L-NAME inhibitor did not promote a shift towards increased arginase activity.

### 3.5. TAMs Stimulated with IFN-γ Plus TLR Agonist Produced IL-10 and TNF-α but No IL-12

**Figure 5:**
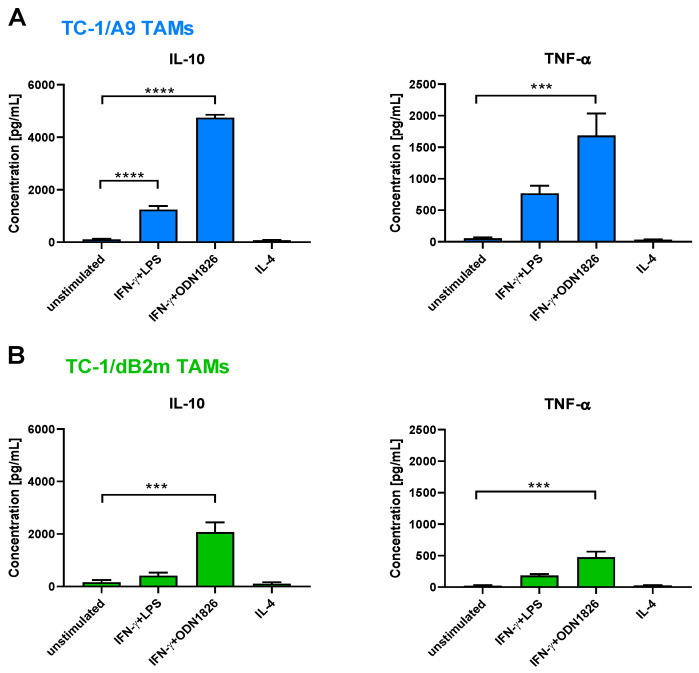
In Vitro production of cytokines by stimulated TAMs. TAMs from TC-1/A9 (**A**) or TC-1/dB2m tumors (**B**) were isolated and stimulated with IFN-γ + TLR agonist or IL-4 for 44 h. Subsequently, the cell culture supernatants were collected and used for the measurement of IL-10, IL-12p70, and TNF-α concentrations by ELISA. The unstimulated cells were used as a negative control. Columns indicate mean of three independent experiments; bars: ± SEM; *** *p* < 0.001, **** *p* < 0.0001.

The differences in the production of IL-10 and IL-12 constitute another macrophage classification system, which we examined for broader TAM characterization. The ratio of IL-12/IL-10 is shifted towards the production of the first cytokine in M1 macrophages, while the production of IL-10 prevails in M2 macrophages [48]. To measure the cytokine concentration in activated macrophages, we stimulated TAMs from TC-1/A9- and TC-1/dB2m-induced tumors and, subsequently, measured the concentrations of IL-10, IL-12p70, and TNF-α in cell culture media by ELISA (Figure 5). Stimulation with an IFN-γ plus TLR agonist, either LPS or ODN1826, resulted in the secretion of IL-10 and TNF-α. Both cytokines were more effectively produced in TAMs isolated from TC-1/A9-induced tumors in comparison to TC-1/dB2m-induced tumors. Unstimulated or IL-4-activated TAMs produced scarce amounts of the analyzed cytokines. We also observed that neither TAMs from TC-1/A9- nor from TC-1/dB2m-induced tumors produced IL-12p70 (data not shown).

### 3.6. CSF-1R Blockade Enhanced the Effect of Combined IT But Only in Tumors with Reversible MHC-I Downregulation

**Figure 6:**
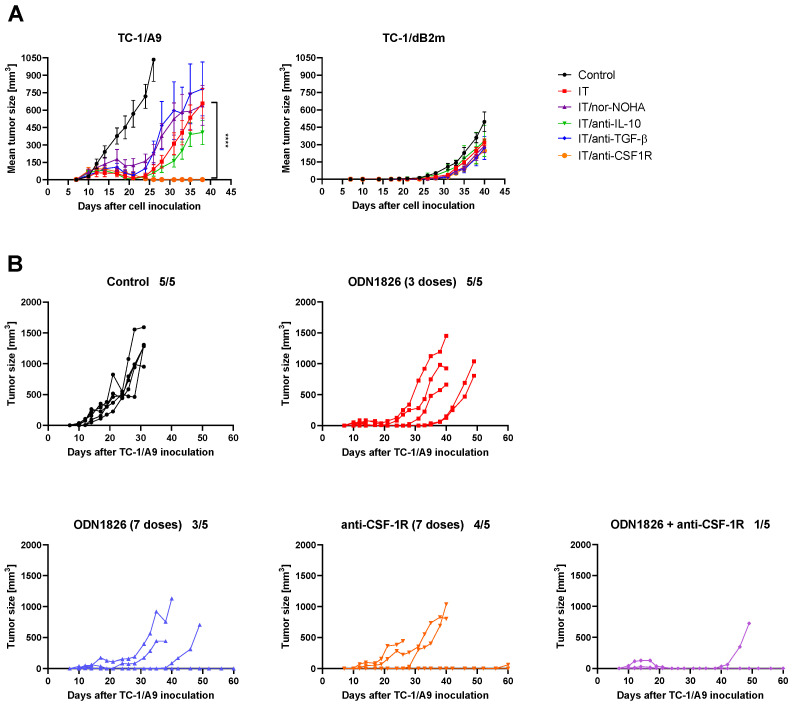
The anti-tumor effect induced by combined IT of tumors with reduced MHC-I expression. (**A**) In Vivo inhibition of immunosuppressive factors. Mice (*n* = 5) were injected with TC-1/A9 or TC-1/dB2m cells and treated with combined IT consisting of DNA immunization and i.p. injection of ODN1826 adjuvant. Arginase activity was inhibited with the nor-NOHA inhibitor, and IL-10, TGF-β, or CSF-1R were neutralized with monoclonal antibodies, as described in Materials and Methods. Bars: ± SEM; **** *p* < 0.0001. Statistical significance refers to the comparison with the group treated with DNA immunization and ODN1826. (**B**) Tumor growth in single mice after different variants of ODN1826 and/or anti-CSF-1R administration. Mice (*n* = 5) were injected with TC-1/A9 cells and immunized with a gene gun and subsequently received the ODN1826 adjuvant, anti-CSF-1R, or their combination, as described in Materials and Methods. Number of mice with tumor/number of mice in a group.

As RNA-seq analysis indicated the expression of immunosuppressive factors in the TME of the treated tumors (Figure S4), we aimed to neutralize those with the highest expression levels, which could have affected the efficacy of the treatment. We attempted to enhance the therapeutic effect of combined IT by neutralizing IL-10, TGF-β, or CSF-1R with monoclonal antibodies. Additionally, we applied nor-NOHA to inhibit the arginase activity in tumors (Figure 6A).

Of the variants tested, only the blockade of CSF-1R significantly enhanced the effect of the treatment, resulting in the prolonged regression of TC-1/A9 tumors in comparison to the tumors treated with the original combined IT. Nevertheless, this effect was temporary, as, after day 40, some tumors started to grow out. Moreover, none of the neutralizing antibodies, nor the arginase inhibitor, supported combined IT against the TC-1/dB2m-induced tumors (Figure 6A). As anti-CSF-1R only enhanced the efficacy of combined IT against TC-1/A9 tumors, we next investigated whether a similar tumor growth inhibition could be achieved in this tumor type, using the same dose number of either ODN1826 or anti-CSF-1R, applied separately into the immunized mice. Therefore, we compared our standard administration schedule of ODN1826 (i.e., three doses) with injections of seven doses of either ODN1826 or anti-CSF-1R (Figure 6B). The administration of additional doses of ODN1826 caused delayed tumor growth or complete tumor regression in some mice in comparison to those treated with three doses, and this effect was similar to that caused by anti-CSF-1R therapy. Combined IT with ODN1826 and anti-CSF-1R induced the highest anti-tumor effect when a tumor developed in only one of five mice in 60 days.

In summary, the anti-tumor effect was most profound in mice with TC-1/A9 tumors that were treated with combined IT supplemented with anti-CSF-1R.

### 3.7. Anti-CSF-1R Barely Affected TAM Repolarization and Slightly Reduced Proportion of CSF-1R-Expressing TAMs in Tumors with Reduced MHC-I Expression

As the blockade of CSF-1R has been shown to deplete TAMs [26], or to repolarize them to an M1 anti-tumor phenotype [23,24,25], we next assessed the effects of anti-CSF-1R on TAMs in tumors with reduced MHC-I expression by flow cytometry. To this end, we injected mice with tumor cells and applied our standard schedule of combined IT, consisting of DNA immunization and three or eight doses of ODN1826 adjuvant for TC-1/A9- or TC-1/dB2m-induced tumors, respectively. Anti-CSF-1R was applied 3 days before the first dose of ODN1826 and on the subsequent days after adjuvant injection. Next, untreated tumors were harvested on days 12 or 33 for TC-1/A9 or TC-1/dB2m tumors, respectively, while the treated tumors were harvested on day 2 after the completion of combined IT. At that time, mice with treated TC-1/A9 tumors received four doses of anti-CSF-1R, while those with TC-1/dB2m tumors were given eight doses. In contrast to the results presented in Figure 2, we performed TAM analysis only 2 days post-treatment, as most tumors in mice with TC-1/A9 tumors, treated with combined IT plus anti-CSF-1R, completely regressed at a later stage.

**Figure 7:**
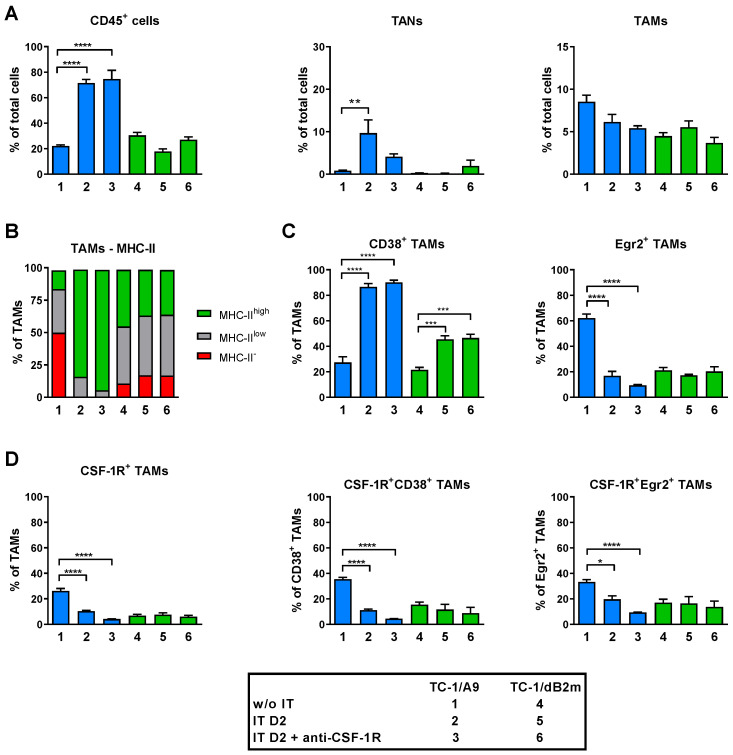
The effects of anti-CSF-1R on polarization and proportion of TAMs in tumors with downregulated MHC-I expression. Mice were injected with tumor cells and subsequently treated with combined IT, consisting of DNA immunization by a gene gun, and i.p. injections of the ODN1826 adjuvant and monoclonal antibody anti-CSF-1R, as described in Material and Methods. On day 2 (D2) after completion of treatment, cells were isolated from tumors, and myeloid cells were analyzed by flow cytometry. Non-treated tumors were used as a control. (**A**) Infiltration of tumors with immune cells (CD45^+^), TANs (CD11b^+^Ly6G^high^Ly6C^low^), and TAMs (CD11b^+^Ly6G^−^Ly6C^−^F4/80^+^). (**B**) Overview of the mean percentages of TAM subpopulations, as determined by MHC-II expression. (**C**) Analysis of M1 and M2 TAM frequencies using CD38 and Egr2 markers, respectively. (**D**) Frequencies of TAMs expressing CSF-1R. Bars: ± SEM; * *p* < 0.05, ** *p* < 0.01, *** *p* < 0.001, **** *p* < 0.0001.

We observed that the enhancement of combined IT with anti-CSF-1R did not cause any significant changes in tumor infiltration with immune cells (CD45^+^) in both TC-1/A9 and TC-1/dB2m tumors. The proportion of the TAN population in the treated TC-1/A9 tumors slightly decreased with the addition of anti-CSF-1R, but the number of TAMs was not significantly affected by this monoclonal antibody (Figure 7A).

Additionally, minor changes in the polarization of TAMs to the M1 phenotype, as indicated by MHC-II expression, were found in TC-1/A9 tumors after anti-CSF-1R injections (Figure 7B). Similarly, the proportion of CD38^+^ TAMs was not altered after the addition of anti-CSF-1R in both tumor types in comparison with the original IT, and the number of Egr2^+^ TAMs only marginally decreased after the addition of anti-CSF-1R into treated mice with TC-1/A9 tumors (Figure 7C). The proportion of TAMs expressing CSF-1R in TC-1/A9 tumors significantly decreased after combined IT and was further reduced in the anti-CSF-1R supplementation group but remained unchanged in TC-1/dB2m tumors (Figure 7D). Considering the expression of CSF-1R on the individual M1 and M2 TAM subpopulations, distinguished by the CD38/Egr2 classification, we found that the proportion of CSF-1R^+^ TAMs decreased in both TAM types in TC-1/A9 tumors, both after the original combined IT and IT supplemented with anti-CSF-1R (Figure 7D).

Taken together, these data show that the addition of anti-CSF-1R into combined IT did not significantly change the infiltration of tumors with immune cells, including TAMs, but reduced the proportion of TAMs expressing CSF-1R in both M1 and M2 macrophages.

## 4. Discussion

Since Hanahan and Weinberg enlarged the spectrum of cancer hallmarks with the ability of a tumor to escape immune surveillance [49], it has become obvious that novel immunotherapies should focus on targeting both tumor cells and the TME. No other cell type is more controversial with respect to immunotherapeutic modulation within the TME than TAMs. Depending on the tumor type, these cells abundantly populate tumor stroma and parenchyma, and, due to their ability to adjust to the signals from the surroundings, their alteration could be applied as an immunotherapeutic strategy.

In previous studies, we investigated the combined IT of tumors with reversible [15] and irreversible downregulation of MHC-I molecules [29] and identified cell populations contributing to the anti-tumor response in the treated TC-1/A9- and TC-1/dB2m-induced tumors, where either temporal regression or delayed tumor growth was observed, respectively. Additionally, we demonstrated that TAMs comprised the major subpopulation of the myeloid compartment in both tumor types, but they only contributed to the anti-tumor effect of combined IT in TC-1/A9-induced tumors. In the present study, we tested the differences between the TMEs of TC-1/A9- and TC-1/dB2m-induced tumors in more detail, focused on the TAM phenotype and function, and attempted to enhance an anti-tumor effect of combined IT.

First, we examined anti-tumor immunity in the treated tumors at different stages of tumor development. For this reason, we isolated RNA and cells from TC-1, TC-/A9, and TC-1/dB2m naïve tumors and tumors 2 and 9 days after completion of combined IT, which encompassed DNA immunization against the HPV16 E7 oncoprotein and i.p. injections of the ODN1826 adjuvant. Unmethylated CpG ODN1826, a TLR9 agonist, has been shown to affect tumor growth in TC-1- and TC-1/A9-induced tumors [50] and has also been demonstrated to be an effective adjuvant for DNA immunization in the TC-1 tumor model [51].

RNA-seq analysis showed the high activation of innate and adaptive immune reactions in TC-1 and TC-1/A9 tumors after IT, but a very low alteration in gene expression in the TME of TC-1/dB2m tumors. Nine days after IT completion, the expression profile in TC-1 and TC-1/A9 tumors resembled that of naïve tumors. The analysis of gene expression also suggested a similarity of TAMs and their alterations after IT in TC-1 and TC-1/A9 tumors, while TAMs in TC-1/dB2m tumors had different expression characteristics, which did not markedly change after IT. However, the number of immune cells infiltrating naïve tumors was similar for all three tumor types. We only found a few differences in the polarization of TAMs and the proportions of lymphoid and myeloid cell subpopulations in TC-1/dB2m tumors in our previous study [29]. Thus, the different TME in TC-1/dB2m tumors was most evident 2 days after IT when, in TC-1 and TC-1/A9 tumors, the expression profile was profoundly altered, the numbers of infiltrating immune cells were greatly increased, and TAMs were polarized into the M1 phenotype, while only subtle modifications were recorded in TC-1/dB2m tumors. The lack of response in the TC-1/dB2m TME, including TAMs, is probably primarily caused by the deactivation of β-2 microglobulin in TC-1/dB2m cells, leading to the loss of antigen presentation by MHC-I molecules and a “cold” or immunosuppressed TME [52].

Flow cytometry analysis of the tumor-infiltrating myeloid cells revealed that TAMs from tumors with distinct MHC-I expression could acquire and maintain an M1 phenotype upon accomplishment of combined IT. In contrast to our previous studies, we enriched our flow cytometry panel for the analysis of myeloid cells with recently described CD38/Egr2 markers, which were used to characterize M1/M2 murine macrophages [43]. The use of this classification system provided us with better insight into the dynamics of TAM polarization in comparison to the MHC-II marker, particularly in TC-1/dB2m-induced tumors, where TAMs expressed high levels of MHC-II molecules, irrespective of the treatment and time of the post-treatment analysis, which is consistent with our previous report [29]. Recent studies have demonstrated that both MHC-II^low^ and MHC-II^high^ TAMs can be found in various tumor types [16,17], indicating that the accumulation of TAMs, expressing variable levels of MHC-II molecules, is TME-intrinsic. By means of CD38/Egr2 classification, we observed that, in all tumor types, the proportion of M2 TAMs declined after IT, while that of M1 TAMs increased in TC-1- and TC-1/A9-treated tumors. In the treated TC-1- and TC-1/A9-induced tumors, TAM polarization towards the M1 phenotype seemed to be transient, as it was maximized 2 days after treatment with a subsequent decline recorded 1 week later.

Co-culture experiments demonstrated that TAMs isolated from tumors with distinct MHC-I surface expression exhibited a different potential for tumor cell killing. While TAMs from TC-1/A9 tumors, which were stimulated with an IFN-γ + TLR agonist to the anti-tumor M1 phenotype, inhibited the proliferation of TC-1/A9 cells, TAMs from the TC-1/dB2m tumors hardly affected the proliferation of TC-1/dB2m cells. The anti-tumor effect of TC-1/A9 TAMs was mediated by both NO and TNF-α, which is in line with previously published reports of the cytotoxic potential of macrophages [18,44]. As TC-1/dB2m TAMs were unable to generate TNF-α and NO in concentrations as high as those of TC-1/A9 TAMs, their cytotoxicity might have been limited. Alternatively, TC-1/dB2m cells may have become resistant to TAM-mediated killing. The varying sensitivity of cell lines towards NO has already been described [44,53,54]. To better understand the involved mechanism, we investigated the interactions of both TAM types with TC-1 cells, where co-cultures were stimulated with IFN-γ + ODN1826, and NO production with its corresponding cytotoxicity was measured. We found out that both types of TAMs showed the same capability for NO production and subsequent cytotoxicity against TC-1 target cells as TAMs co-cultured with tumor cell lines used in the initial experiment. Hence, we concluded that the differing capabilities of TAMs from TC-1/A9 and TC-1/dB2m tumors for NO and TNF-α release, and the related cytotoxicity, are independent of the used target cells but are TAM-inherent. These results also highlight the importance of the NO concentration necessary for tumor cell killing. While low NO quantities may support tumor growth or proliferation, high concentrations can destroy tumor cells [55].

One of the focuses of this study was on arginine metabolism in both types of TAMs. In vitro, we found that blocking iNOS activity with the L-NAME inhibitor in TAMs, stimulated to the M1 phenotype with IFN-γ + ODN1826, did not promote an increase in arginase activity, although the arginine pool was larger for use with arginase. On the other hand, in our experimental setup, iNOS activity was not fully blocked, but rather decreased; thus, NO was still produced. A recent study conducted on bone marrow-derived macrophages demonstrated that high NO production by M1 macrophages that were stimulated with IFN-γ + LPS caused irreversible changes in mitochondria and thus disabled the upregulation of M2-associated genes when the cells were subsequently stimulated with IL-4. The only gene with unaffected expression was the arginase 1 gene, although arginase enzymatic activity was significantly lower after repolarization [56]. In contrast to these findings, arginase activity in our experimental setup was comparable in M1 and L-NAME-treated M1 TAMs, suggesting that arginine availability in the latter case was still insufficient for the increased use of arginine by arginase. Secondly, we did not observe the enhancement of the anti-tumor effect with the arginase inhibitor nor-NOHA in the treated tumors with downregulated MHC-I expression, despite the fact that arginase-producing TAMs were present in the TME, particularly in TC-1/A9 tumors. This result is in contrast with the data obtained for lung carcinoma induced with 3LL cells, where treatment with nor-NOHA inhibited tumor growth [57]. Taken together, the results from in vitro and in vivo targeting of arginine-consuming enzymes suggest that treatment affecting arginine metabolism in tumors with reduced MHC-I expression may not have a significant impact on the enhancement of combined IT.

To our surprise, TAMs stimulated with the IFN-γ + TLR agonist did not produce IL-12, which is considered a hallmark of M1 macrophages. In our experimental setup, we chose to measure IL-12p70, as this form of cytokine is bioactive, while the measurement of its subunits, p35 or p40, would indicate the presence of other interleukins with antagonistic activities with regard to the tumor IT [58]. We speculate that TAMs isolated from tumors with reduced MHC-I expression did not produce IL-12p70 because of the presence of high concentrations of IL-10, which could counterbalance IL-12 production, as demonstrated on the RAW 264.7 macrophage cell line [59]. On the other hand, the study by Banerjee and colleagues showed that the differences in IL-12 production may lie in both the heterogeneity of macrophages and stimulus used. While LPS-activated TAMs from B16 melanoma produced small amounts of IL-12, macrophages activated with heat-killed *Mycobacterium* produced large amounts of this cytokine. At the same time, peritoneal macrophages secreted high amounts of IL-12, irrespective of stimulation [60].

RNA sequencing revealed that certain immunosuppressive factors were expressed in the TME of both tumor types with decreased MHC-I expression after treatment with DNA immunization and the ODN1826 adjuvant. Hence, we aimed for an improvement in the anti-tumor effect of combined IT by injecting either monoclonal antibodies to neutralize IL-10, TGF-β, and CSF-1R or the nor-NOHA inhibitor to block the activity of arginase. Neither the neutralization of IL-10 or TGF-β nor the inhibition of arginase resulted in the enhancement of our combined IT. These observations differ from studies conducted in tumors induced with parental TC-1 cells, as targeting either IL-10 or TGF-β enhanced the therapeutic effect of various immunotherapies or increased infiltration with an anti-tumor cell population in these tumors [61,62,63]. This difference can be caused by the higher resistance of tumors with MHC-I downregulation to IT and the different mechanisms of tumor cell killing.

We observed that the blockade of CSF-1R resulted in the marked enhancement of combined IT, but only in TC-1/A9 tumors, which could be connected with the involvement of TAMs in the IT of this tumor type [15]; in TC-1/dB2m tumors, the anti-tumor effect of TAMs was not proved [29]. The inhibition of CSF-1/CSF-1R signaling, either with a monoclonal antibody or small molecule inhibitor, yielded encouraging results in preclinical studies, but the outcomes of clinical trials have not been satisfactory [13]. Several mechanisms have been suggested which could contribute to this failure, mainly a compensatory intratumoral increase in the number of FoxP3^+^ Treg cells [64] or polymorphonuclear/granulocytic MDSCs [65,66]. However, in TC-1/dB2m tumors, TAMs are probably blocked in an unresponsiveness state and cannot be polarized into a phenotype with anti-tumor capability. β-2 microglobulin-free classical MHC-I heavy chains, which are exhibited on TC-1/dB2m cells after IFN-γ stimulation [29], could contribute to the establishment of this state by binding with TAM receptors [67].

Additionally, we have demonstrated that the combination of ODN1826 and anti-CSF-1R was crucial for the impact of IT on TC-1/A9-induced tumors. This result resembles the efficacy of anti-CSF-1R treatment in combination with other ITs, e.g., while anti-CSF-1R was inefficient as a monotherapy, it supported the anti-tumor effect of the anti-CD40 monoclonal antibody in murine colon tumor models [25]. In another study, anti-CSF-1R significantly improved the anti-tumor effect of combined IT, consisting of anti-CTLA and anti-PD-L1, in pancreatic carcinoma models [23].

Different effects of CSF-1R blockade on TAMs have been reported. While the small molecule inhibitor PLX3397 did not influence the number of infiltrating TAMs but reprogrammed them to the M1 phenotype in tumors induced with various human hepatocellular carcinoma cell lines [24], the same compound caused the depletion of both M1 and M2 TAMs in a murine mesothelioma tumor model and thus enhanced the anti-tumor effect of dendritic cell-based vaccination [68]. Another study showed that treatment with anti-CSF-1R resulted in the marked depletion of M2 TAMs from the TME of pancreatic carcinoma with the subsequent repolarization of the remaining TAMs to the M1 phenotype [23]. In our study, we did not observe significant differences between phenotypes of TAMs from tumors treated with either original combined IT or combined IT supplemented with anti-CSF-1R administration. However, we observed that anti-CSF-1R reduced the frequency of CSF-1R^+^ TAMs in TC-1/A9 tumors, and Egr2^+^ TAMs seemed to be more prone to this depletion. This observation is in line with previously published results showing that anti-CSF-1R preferentially depleted CD206^high^ TAMs which, similar to Egr2^+^ TAMs, are representative of the M2 phenotype [23]. Moreover, TAM subpopulations with different sensitivities to the CSF-1R blockade have been characterized by single-cell RNA-seq [26,66], suggesting that TAM heterogeneity may be a critical parameter when considering anti-CSF-1R therapy. Finally, CSF-1R blockade not only influences the TAM presence in the TME but can also promote CD8^+^ T cell infiltration and/or decrease FoxP3^+^ Tregs, MDSCs, and CD4^+^ T cells in tumors, depending on the type of tumor model [23,24,25], which opens a new area of research for investigation of an anti-CSF-1R therapy in tumors with a distinct MHC-I expression.

## 5. Conclusions

In this study, we demonstrated that the TMEs in tumors with a distinct MHC-I expression are modulated to different extents by combined IT. While tumors with reversible MHC-I downregulation were sensitive to treatment, tumors characterized by irreversible MHC-I expression were affected, to a lesser extent, by combined IT. Similarly, TAMs isolated from these tumors showed distinct capabilities for repolarization to the anti-tumor M1 phenotype, as evidenced by their ex vivo phenotypic characterization and in vitro ability to inhibit tumor cell proliferation. Correspondingly, the CSF-1R blockade markedly enhanced the effect of combined IT, but only in tumors with a reversible reduction in the number of MHC-I molecules. Detailed studies must be undertaken to examine the impact of anti-CSF-1R on TAMs and its role in combined IT of tumors with varying levels of MHC-I expression.

## Figures and Tables

**Table 1:** List of antibodies used for flow cytometry.

Antigen	Conjugate	Clone	Company	Staining	Panels	
Arg-1	APC	A1exF5	eBioscience	Intracellular	•	
CD11b	BV421	M1/70	BioLegend	Surface	•	•
CD11c	APC-Cy7	N418	BioLegend	Surface	•	•
CD38	BV605	90	BD Biosciences	Surface	•	•
CD45	AF700	30-F11	BioLegend	Surface	•	•
CSF-1R	PerCP-eFluor710	AFS98	eBioscience	Surface		•
Egr2	PE	erongr2	eBioscience	Intracellular	•	•
F4/80	BV650	BM8	BioLegend	Surface	•	•
IDO1	PerCP-eFluor710	mIDO-48	eBioscience	Intracellular	•	
iNOS	AF488	CXNFT	eBioscience	Intracellular	•	
Ly6C	BV785	HK1.4	BioLegend	Surface	•	•
Ly6G	PE-Cy5	1A8	Reagent Genie	Surface	•	•
MHC-II	PE-Cy7	M5/114.15.2	BioLegend	Surface	•	•
TNF-α	PE/Dazzle594	MP6-XT22	BioLegend	Intracellular	•	

## Data Availability

The RNA-seq data were deposited in the NCBI Sequence Read Archive, BioProject accession number PRJNA714232.

## Supplementary Materials

The following are available online at https://www.mdpi.com/article/10.3390/cancers13123057/s1, Figure S1: Gating strategy for flow cytometry analysis of Figure 2, Figure S2: Gating strategy for flow cytometry analysis of Figure 7, Figure S3: Differential gene expression, Figure S4: Cluster analysis of immune-related genes. Table S1: Immune-related genes.

## References

[1] Thor Straten P., Garrido F. (2016). Targetless T Cells in Cancer Immunotherapy. J. Immunother. Cancer.

[2] Najafimehr H., Hajizadeh N., Nazemalhosseini-Mojarad E., Pourhoseingholi M.A., Abdollahpour-Alitappeh M., Ashtari S., Zali M.R. (2020). The Role of Human Leukocyte Antigen Class I on Patient Survival in Gastrointestinal Cancers: A Systematic Review and Meta-Analysis. Sci. Rep..

[3] Schaafsma E., Fugle C.M., Wang X., Cheng C. (2021). Pan-Cancer Association of HLA Gene Expression with Cancer Prognosis and Immunotherapy Efficacy. Br. J. Cancer.

[4] Ryschich E., Nötzel T., Hinz U., Autschbach F., Ferguson J., Simon I., Weitz J., Fröhlich B., Klar E., Büchler M.W. (2005). Control of T-Cell–Mediated Immune Response by HLA Class I in Human Pancreatic Carcinoma. Clin. Cancer Res..

[5] Goeppert B., Frauenschuh L., Zucknick M., Roessler S., Mehrabi A., Hafezi M., Stenzinger A., Warth A., Pathil A., Renner M. (2015). Major Histocompatibility Complex Class I Expression Impacts on Patient Survival and Type and Density of Immune Cells in Biliary Tract Cancer. Br. J. Cancer.

[6] Perea F., Bernal M., Sánchez-Palencia A., Carretero J., Torres C., Bayarri C., Gómez-Morales M., Garrido F., Ruiz-Cabello F. (2017). The Absence of HLA Class I Expression in Non-Small Cell Lung Cancer Correlates with the Tumor Tissue Structure and the Pattern of T Cell Infiltration. Int. J. Cancer.

[7] Garrido F., Perea F., Bernal M., Sánchez-Palencia A., Aptsiauri N., Ruiz-Cabello F. (2017). The Escape of Cancer from T Cell-Mediated Immune Surveillance: HLA Class I Loss and Tumor Tissue Architecture. Vaccines.

[8] Singh M., Khong H., Dai Z., Huang X.-F., Wargo J.A., Cooper Z.A., Vasilakos J.P., Hwu P., Overwijk W.W. (2014). Effective Innate and Adaptive Antimelanoma Immunity through Localized TLR7/8 Activation. J. Immunol..

[9] Moynihan K.D., Irvine D.J. (2017). Roles for Innate Immunity in Combination Immunotherapies. Cancer Res..

[10] Rakhmilevich A.L., Felder M., Lever L., Slowinski J., Rasmussen K., Hoefges A., van de Voort T.J., Loibner H., Korman A.J., Gillies S.D. (2017). Effective Combination of Innate and Adaptive Immunotherapeutic Approaches in a Mouse Melanoma Model. J. Immunol..

[11] Hartl C.A., Bertschi A., Puerto R.B., Andresen C., Cheney E.M., Mittendorf E.A., Guerriero J.L., Goldberg M.S. (2019). Combination Therapy Targeting Both Innate and Adaptive Immunity Improves Survival in a Pre-Clinical Model of Ovarian Cancer. J. Immunother. Cancer.

[12] Quaranta V., Schmid M.C. (2019). Macrophage-Mediated Subversion of Anti-Tumour Immunity. Cells.

[13] Kowal J., Kornete M., Joyce J.A. (2019). Re-Education of Macrophages as a Therapeutic Strategy in Cancer. Immunotherapy.

[14] Buhtoiarov I.N., Sondel P.M., Eickhoff J.C., Rakhmilevich A.L. (2007). Macrophages Are Essential for Antitumour Effects against Weakly Immunogenic Murine Tumours Induced by Class B CpG-Oligodeoxynucleotides. Immunology.

[15] Grzelak A., Polakova I., Smahelova J., Vackova J., Pekarcikova L., Tachezy R., Smahel M. (2018). Experimental Combined Immunotherapy of Tumours with Major Histocompatibility Complex Class I Downregulation. Int. J. Mol. Sci..

[16] Movahedi K., Laoui D., Gysemans C., Baeten M., Stangé G., van den Bossche J., Mack M., Pipeleers D., In’t Veld P., de Baetselier P. (2010). Different Tumor Microenvironments Contain Functionally Distinct Subsets of Macrophages Derived from Ly6C (High) Monocytes. Cancer Res..

[17] Wang B., Li Q., Qin L., Zhao S., Wang J., Chen X. (2011). Transition of Tumor-Associated Macrophages from MHC Class II (Hi) to MHC Class II (Low) Mediates Tumor Progression in Mice. BMC Immunol..

[18] Lum H.D., Buhtoiarov I.N., Schmidt B.E., Berke G., Paulnock D.M., Sondel P.M., Rakhmilevich A.L. (2006). Tumoristatic Effects of Anti-CD40 MAb-Activated Macrophages Involve Nitric Oxide and Tumour Necrosis Factor-Alpha. Immunology.

[19] Ellyard J.I., Quah B.J.C., Simson L., Parish C.R. (2010). Alternatively Activated Macrophage Possess Antitumor Cytotoxicity That Is Induced by IL-4 and Mediated by Arginase-1. J. Immunother..

[20] Peranzoni E., Lemoine J., Vimeux L., Feuillet V., Barrin S., Kantari-Mimoun C., Bercovici N., Guérin M., Biton J., Ouakrim H. (2018). Macrophages Impede CD8 T Cells from Reaching Tumor Cells and Limit the Efficacy of Anti-PD-1 Treatment. Proc. Natl. Acad. Sci. USA.

[21] Thoreau M., Penny H.L., Tan K., Regnier F., Weiss J.M., Lee B., Johannes L., Dransart E., Le Bon A., Abastado J.-P. (2015). Vaccine-Induced Tumor Regression Requires a Dynamic Cooperation between T Cells and Myeloid Cells at the Tumor Site. Oncotarget.

[22] Stanley E.R., Chitu V. (2014). CSF-1 Receptor Signaling in Myeloid Cells. Cold Spring Harb. Perspect. Biol..

[23] Zhu Y., Knolhoff B.L., Meyer M.A., Nywening T.M., West B.L., Luo J., Wang-Gillam A., Goedegebuure S.P., Linehan D.C., DeNardo D.G. (2014). CSF1/CSF1R Blockade Reprograms Tumor-Infiltrating Macrophages and Improves Response to T-Cell Checkpoint Immunotherapy in Pancreatic Cancer Models. Cancer Res..

[24] Ao J.-Y., Zhu X.-D., Chai Z.-T., Cai H., Zhang Y.-Y., Zhang K.-Z., Kong L.-Q., Zhang N., Ye B.-G., Ma D.-N. (2017). Colony-Stimulating Factor 1 Receptor Blockade Inhibits Tumor Growth by Altering the Polarization of Tumor-Associated Macrophages in Hepatocellular Carcinoma. Mol. Cancer Ther..

[25] Wiehagen K.R., Girgis N.M., Yamada D.H., Smith A.A., Chan S.R., Grewal I.S., Quigley M., Verona R.I. (2017). Combination of CD40 Agonism and CSF-1R Blockade Reconditions Tumor-Associated Macrophages and Drives Potent Antitumor Immunity. Cancer Immunol. Res..

[26] Zhang L., Li Z., Skrzypczynska K.M., Fang Q., Zhang W., O’Brien S.A., He Y., Wang L., Zhang Q., Kim A. (2020). Single-Cell Analyses Inform Mechanisms of Myeloid-Targeted Therapies in Colon Cancer. Cell.

[27] Lin K.Y., Guarnieri F.G., Staveley-O’Carroll K.F., Levitsky H.I., August J.T., Pardoll D.M., Wu T.C. (1996). Treatment of Established Tumors with a Novel Vaccine That Enhances Major Histocompatibility Class II Presentation of Tumor Antigen. Cancer Res..

[28] Smahel M., Síma P., Ludvíková V., Marinov I., Pokorná D., Vonka V. (2003). Immunisation with Modified HPV16 E7 Genes against Mouse Oncogenic TC-1 Cell Sublines with Downregulated Expression of MHC Class I Molecules. Vaccine.

[29] Lhotakova K., Grzelak A., Polakova I., Vackova J., Smahel M. (2019). Establishment and Characterization of a Mouse Tumor Cell Line with Irreversible Downregulation of MHC Class I Molecules. Oncol. Rep..

[30] Smahel M., Polakova I., Duskova M., Ludvikova V., Kastankova I. (2014). The Effect of Helper Epitopes and Cellular Localization of an Antigen on the Outcome of Gene Gun DNA Immunization. Gene Ther..

[31] Smahel M., Síma P., Ludvíková V., Vonka V. (2001). Modified HPV16 E7 Genes as DNA Vaccine against E7-Containing Oncogenic Cells. Virology.

[32] Alexander J., Sidney J., Southwood S., Ruppert J., Oseroff C., Maewal A., Snoke K., Serra H.M., Kubo R.T., Sette A. (1994). Development of High Potency Universal DR-Restricted Helper Epitopes by Modification of High Affinity DR-Blocking Peptides. Immunity.

[33] Love M.I., Huber W., Anders S. (2014). Moderated Estimation of Fold Change and Dispersion for RNA-Seq Data with DESeq2. Genome Biol..

[34] Chen E.Y., Tan C.M., Kou Y., Duan Q., Wang Z., Meirelles G.V., Clark N.R., Ma’ayan A. (2013). Enrichr: Interactive and Collaborative HTML5 Gene List Enrichment Analysis Tool. BMC Bioinform..

[35] Kuleshov M.V., Jones M.R., Rouillard A.D., Fernandez N.F., Duan Q., Wang Z., Koplev S., Jenkins S.L., Jagodnik K.M., Lachmann A. (2016). Enrichr: A Comprehensive Gene Set Enrichment Analysis Web Server 2016 Update. Nucleic Acids Res..

[36] Angelova M., Charoentong P., Hackl H., Fischer M.L., Snajder R., Krogsdam A.M., Waldner M.J., Bindea G., Mlecnik B., Galon J. (2015). Characterization of the Immunophenotypes and Antigenomes of Colorectal Cancers Reveals Distinct Tumor Escape Mechanisms and Novel Targets for Immunotherapy. Genome Biol..

[37] Charoentong P., Finotello F., Angelova M., Mayer C., Efremova M., Rieder D., Hackl H., Trajanoski Z. (2017). Pan-Cancer Immunogenomic Analyses Reveal Genotype-Immunophenotype Relationships and Predictors of Response to Checkpoint Blockade. Cell Rep..

[38] Thorsson V., Gibbs D.L., Brown S.D., Wolf D., Bortone D.S., Ou Yang T.-H., Porta-Pardo E., Gao G.F., Plaisier C.L., Eddy J.A. (2018). The Immune Landscape of Cancer. Immunity.

[39] Metsalu T., Vilo J. (2015). ClustVis: A Web Tool for Visualizing Clustering of Multivariate Data Using Principal Component Analysis and Heatmap. Nucleic Acids Res..

[40] Kaštánková I., Poláková I., Dušková M., Šmahel M. (2016). Combined Cancer Immunotherapy Against Aurora Kinase A. J. Immunother..

[41] Ferrari M., Fornasiero M.C., Isetta A.M. (1990). MTT Colorimetric Assay for Testing Macrophage Cytotoxic Activity in Vitro. J. Immunol. Methods.

[42] Corraliza I.M., Campo M.L., Soler G., Modolell M. (1994). Determination of Arginase Activity in Macrophages: A Micromethod. J. Immunol. Methods.

[43] Jablonski K.A., Amici S.A., Webb L.M., de Ruiz-Rosado J.D., Popovich P.G., Partida-Sanchez S., Guerau-de-Arellano M. (2015). Novel Markers to Delineate Murine M1 and M2 Macrophages. PLoS ONE.

[44] Routes J.M., Morris K., Ellison M.C., Ryan S. (2005). Macrophages Kill Human Papillomavirus Type 16 E6-Expressing Tumor Cells by Tumor Necrosis Factor Alpha- and Nitric Oxide-Dependent Mechanisms. J. Virol..

[45] Chang C.I., Liao J.C., Kuo L. (1998). Arginase Modulates Nitric Oxide Production in Activated Macrophages. Am. J. Physiol..

[46] Mori M. (2007). Regulation of Nitric Oxide Synthesis and Apoptosis by Arginase and Arginine Recycling. J. Nutr..

[47] Zou S., Wang X., Liu P., Ke C., Xu S. (2019). Arginine Metabolism and Deprivation in Cancer Therapy. Biomed. Pharmacother..

[48] Mosser D.M., Edwards J.P. (2008). Exploring the Full Spectrum of Macrophage Activation. Nat. Rev. Immunol..

[49] Hanahan D., Weinberg R.A. (2011). Hallmarks of Cancer: The next Generation. Cell.

[50] Reinis M., Símová J., Bubeník J. (2006). Inhibitory Effects of Unmethylated CpG Oligodeoxynucleotides on MHC Class I-Deficient and -Proficient HPV16-Associated Tumours. Int. J. Cancer.

[51] Šmahel M., Poláková I., Sobotková E., Vajdová E. (2011). Systemic Administration of CpG Oligodeoxynucleotide and Levamisole as Adjuvants for Gene-Gun-Delivered Antitumor DNA Vaccines. Clin. Dev. Immunol..

[52] Galon J., Bruni D. (2019). Approaches to Treat Immune Hot, Altered and Cold Tumours with Combination Immunotherapies. Nat. Rev. Drug Discov..

[53] Cui S., Reichner J.S., Mateo R.B., Albina J.E. (1994). Activated Murine Macrophages Induce Apoptosis in Tumor Cells through Nitric Oxide-Dependent or -Independent Mechanisms. Cancer Res..

[54] Tate D.J., Patterson J.R., Velasco-Gonzalez C., Carroll E.N., Trinh J., Edwards D., Aiyar A., Finkel-Jimenez B., Zea A.H. (2012). Interferon-Gamma-Induced Nitric Oxide Inhibits the Proliferation of Murine Renal Cell Carcinoma Cells. Int. J. Biol. Sci..

[55] Rahat M.A., Hemmerlein B. (2013). Macrophage-Tumor Cell Interactions Regulate the Function of Nitric Oxide. Front. Physiol..

[56] Van den Bossche J., Baardman J., Otto N.A., van der Velden S., Neele A.E., van den Berg S.M., Luque-Martin R., Chen H.-J., Boshuizen M.C.S., Ahmed M. (2016). Mitochondrial Dysfunction Prevents Repolarization of Inflammatory Macrophages. Cell Rep..

[57] Rodriguez P.C., Quiceno D.G., Zabaleta J., Ortiz B., Zea A.H., Piazuelo M.B., Delgado A., Correa P., Brayer J., Sotomayor E.M. (2004). Arginase I Production in the Tumor Microenvironment by Mature Myeloid Cells Inhibits T-Cell Receptor Expression and Antigen-Specific T-Cell Responses. Cancer Res..

[58] Mirlekar B., Pylayeva-Gupta Y. (2021). IL-12 Family Cytokines in Cancer and Immunotherapy. Cancers.

[59] Rahim S.S., Khan N., Boddupalli C.S., Hasnain S.E., Mukhopadhyay S. (2005). Interleukin-10 (IL-10) Mediated Suppression of IL-12 Production in RAW 264.7 Cells Also Involves c-Rel Transcription Factor. Immunology.

[60] Banerjee S., Halder K., Ghosh S., Bose A., Majumdar S. (2015). The Combination of a Novel Immunomodulator with a Regulatory T Cell Suppressing Antibody (DTA-1) Regress Advanced Stage B16F10 Solid Tumor by Repolarizing Tumor Associated Macrophages in Situ. Oncoimmunology.

[61] Chen S., Wang X., Wu X., Wei M.Q., Zhang B., Liu X., Wang Y. (2014). IL-10 Signalling Blockade at the Time of Immunization Inhibits Human Papillomavirus 16 E7 Transformed TC-1 Tumour Cells Growth in Mice. Cell Immunol..

[62] Bialkowski L., van der Jeught K., Bevers S., Tjok Joe P., Renmans D., Heirman C., Aerts J.L., Thielemans K. (2018). Immune Checkpoint Blockade Combined with IL-6 and TGF-β Inhibition Improves the Therapeutic Outcome of MRNA-Based Immunotherapy. Int. J. Cancer.

[63] Chu X., Li Y., Huang W., Feng X., Sun P., Yao Y., Yang X., Sun W., Bai H., Liu C. (2018). Combined Immunization against TGF-Β1 Enhances HPV16 E7-Specific Vaccine-Elicited Antitumour Immunity in Mice with Grafted TC-1 Tumours. Artif. Cells Nanomed. Biotechnol..

[64] Gyori D., Lim E.L., Grant F.M., Spensberger D., Roychoudhuri R., Shuttleworth S.J., Okkenhaug K., Stephens L.R., Hawkins P.T. (2018). Compensation between CSF1R+ Macrophages and Foxp3^+^ Treg Cells Drives Resistance to Tumor Immunotherapy. JCI Insight.

[65] Kumar V., Donthireddy L., Marvel D., Condamine T., Wang F., Lavilla-Alonso S., Hashimoto A., Vonteddu P., Behera R., Goins M.A. (2017). Cancer-Associated Fibroblasts Neutralize the Anti-Tumor Effect of CSF1 Receptor Blockade by Inducing PMN-MDSC Infiltration of Tumors. Cancer Cell.

[66] Loeuillard E., Yang J., Buckarma E., Wang J., Liu Y., Conboy C., Pavelko K.D., Li Y., O’Brien D., Wang C. (2020). Targeting Tumor-Associated Macrophages and Granulocytic Myeloid-Derived Suppressor Cells Augments PD-1 Blockade in Cholangiocarcinoma. J. Clin. Investig..

[67] Marchesi M., Andersson E., Villabona L., Seliger B., Lundqvist A., Kiessling R., Masucci G.V. (2013). HLA-Dependent Tumour Development: A Role for Tumour Associate Macrophages?. J. Transl. Med..

[68] Dammeijer F., Lievense L.A., Kaijen-Lambers M.E., van Nimwegen M., Bezemer K., Hegmans J.P., van Hall T., Hendriks R.W., Aerts J.G. (2017). Depletion of Tumor-Associated Macrophages with a CSF-1R Kinase Inhibitor Enhances Antitumor Immunity and Survival Induced by DC Immunotherapy. Cancer Immunol. Res..

