# Supplementary material for: Distinct Responsiveness of Tumor-Associated Macrophages to Immunotherapy of Tumors with Different Mechanisms of Major Histocompatibility Complex Class I Downregulation
Source: Cancers (Basel). 2021 Jun 19;13(12):3057. doi: 10.3390/cancers13123057 (PMC8235485; doi:10.3390/cancers13123057)
Supplement: Supplementary file 1 [file cancers-13-03057-s001.zip › cancers-1228078-supplementary.pdf]

cancers

Supplementary Materials

Distinct Responsiveness of Tumor-Associated Macrophages to Immunotherapy of Tumors with Different Mechanisms of Major Histocompatibility Complex Class I Downregulation

Adrianna Piatakova, Ingrid Polakova, Jana Smahelova, Shweta Dilip Johari, Jaroslav Nunvar and Michal Smahel

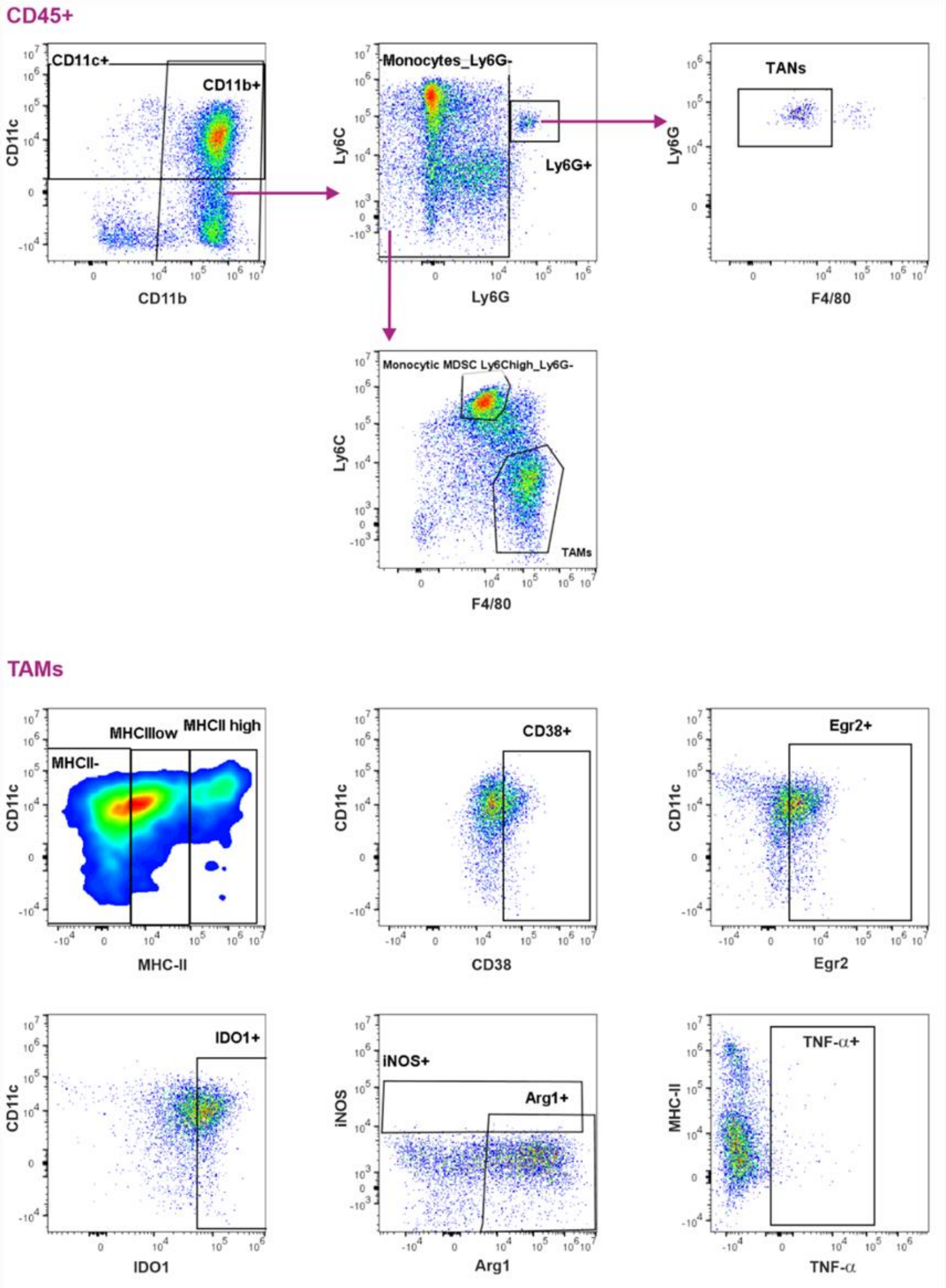

Figure S1. Gating strategy for flow cytometry analysis of Figure 2. Myeloid cells, originating from treated and non-treated tumors (see Figure 2), were identified with flow cytometry analysis, as described in Materials and Methods. The initial gates determined single cells (FSC-A vs. FSC-W) and live cells. The gate on FSC-A vs. SSC-A selected the cells from the tumor, which were divided into CD45$^-$ and CD45$^+$ cells. Populations downstream from CD45$^+$ cells are indicated in the above figure. CD11b, Ly6G, Ly6C and F4/80 markers defined TANs and TAMs. Subpopulations of TAMs expressing MHC-II and the CD38 and Egr2 macrophage markers are also depicted in the figure, followed by subpopulations expressing the amino acid degrading enzymes IDO1, iNOS, and Arg1, and the cytokine TNF-α. The accurate identification of some subpopulations required FMO controls.

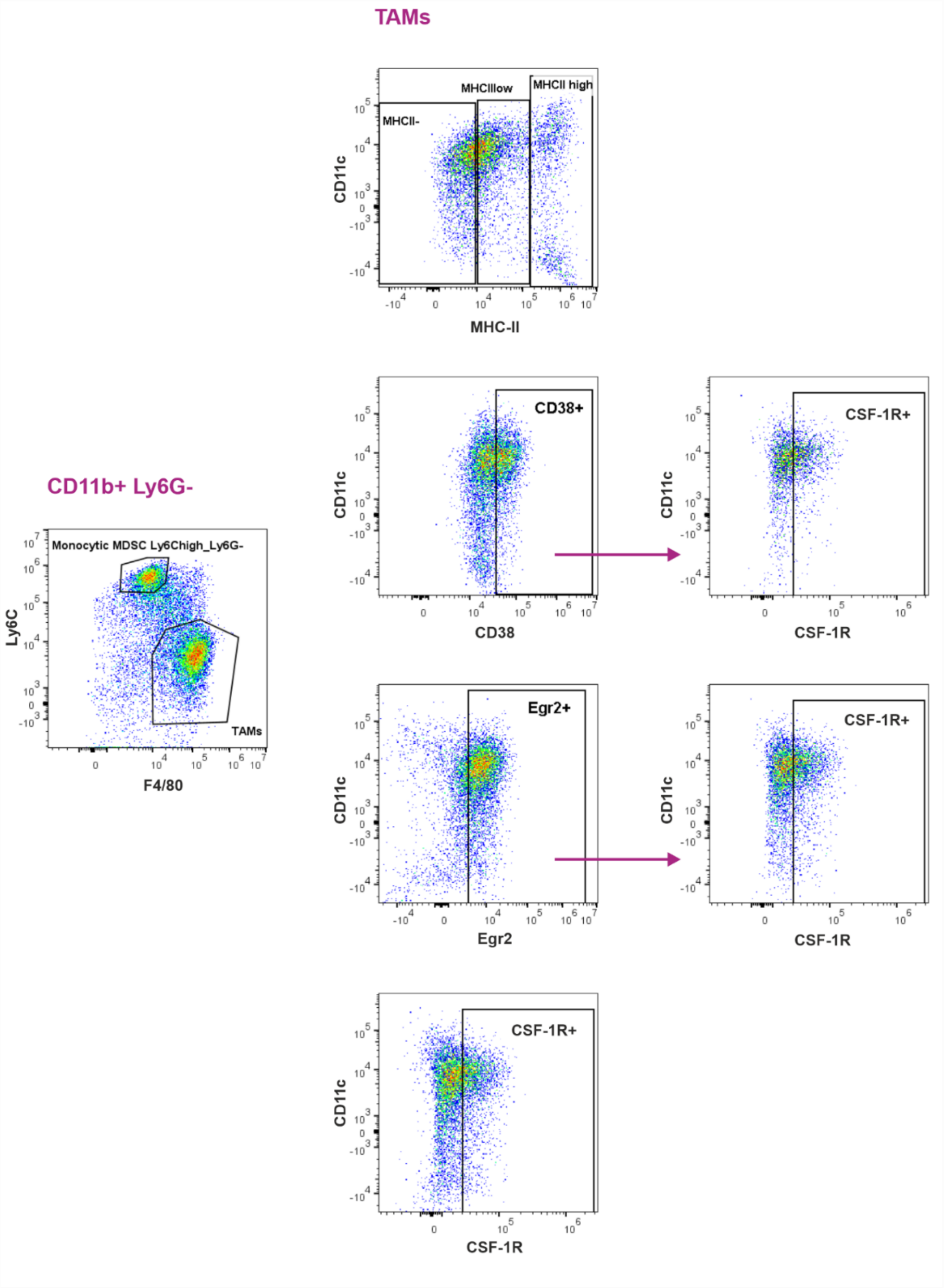

Figure S2. Gating strategy for flow cytometry analysis of Figure 7. TAMs originated from tumors described in Figure 7 were identified using different flow cytometry panel of antibodies, but the same upstream gating hierarchy as delineated in Figure S1. Aside from MHC-II, CD38 and Egr2 macrophage markers, CSF-1R was gated. The arrows indicate CSF-1R-expression on $CD38^{+}$ and $Egr2^{+}$ TAMs. FMO controls were used, if needed.

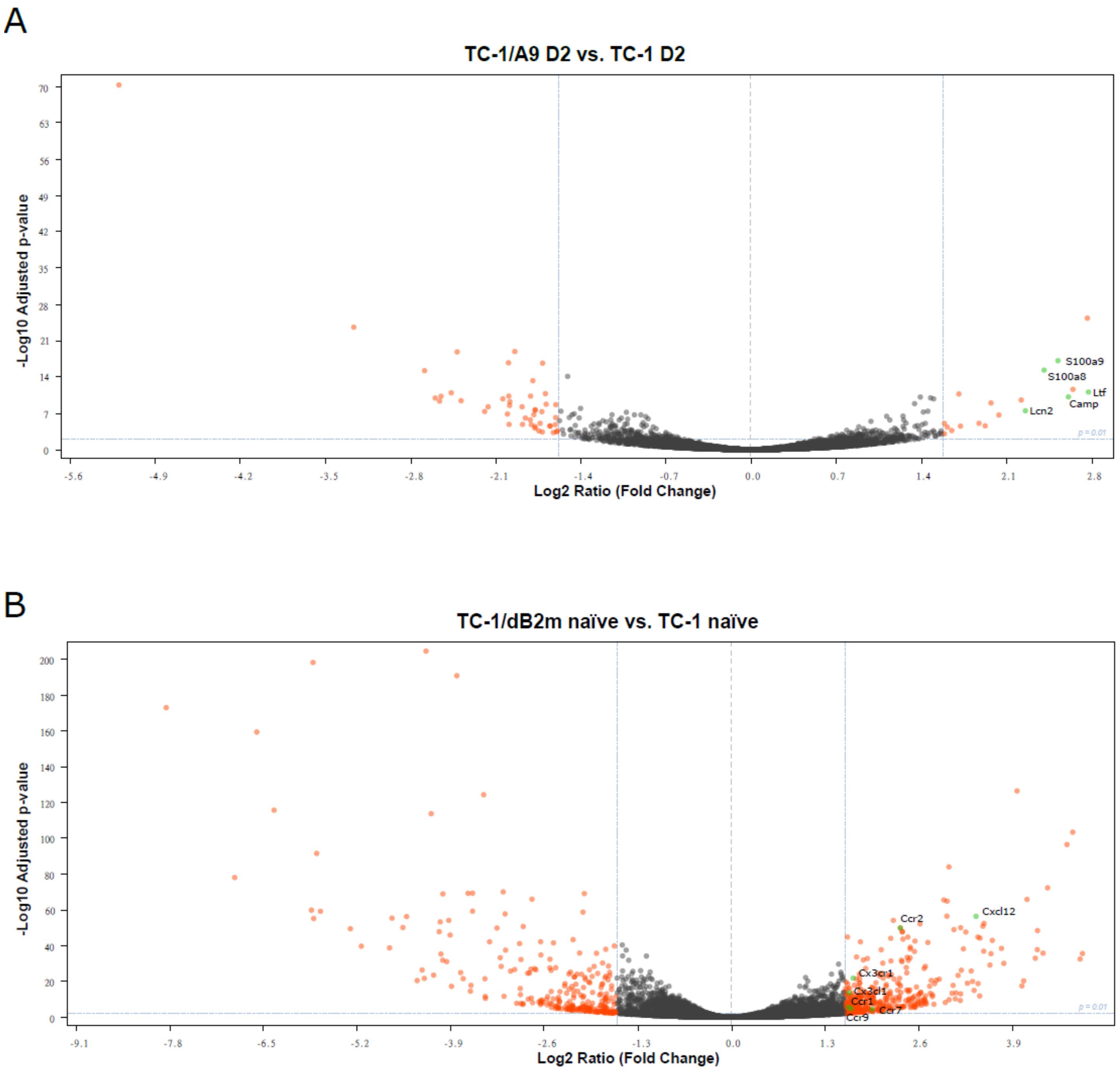

Figure S3: Differential gene expression. (A) Comparison of TC-1/A9 tumors with TC-1 tumors 2 days after IT. Upregulated genes associated with neutrophil activation and degranulation are indicated. (B) Comparison of naïve TC-1/dB2m tumors with TC-1 tumors. Upregulated genes encoding chemokines and their receptors are indicated.

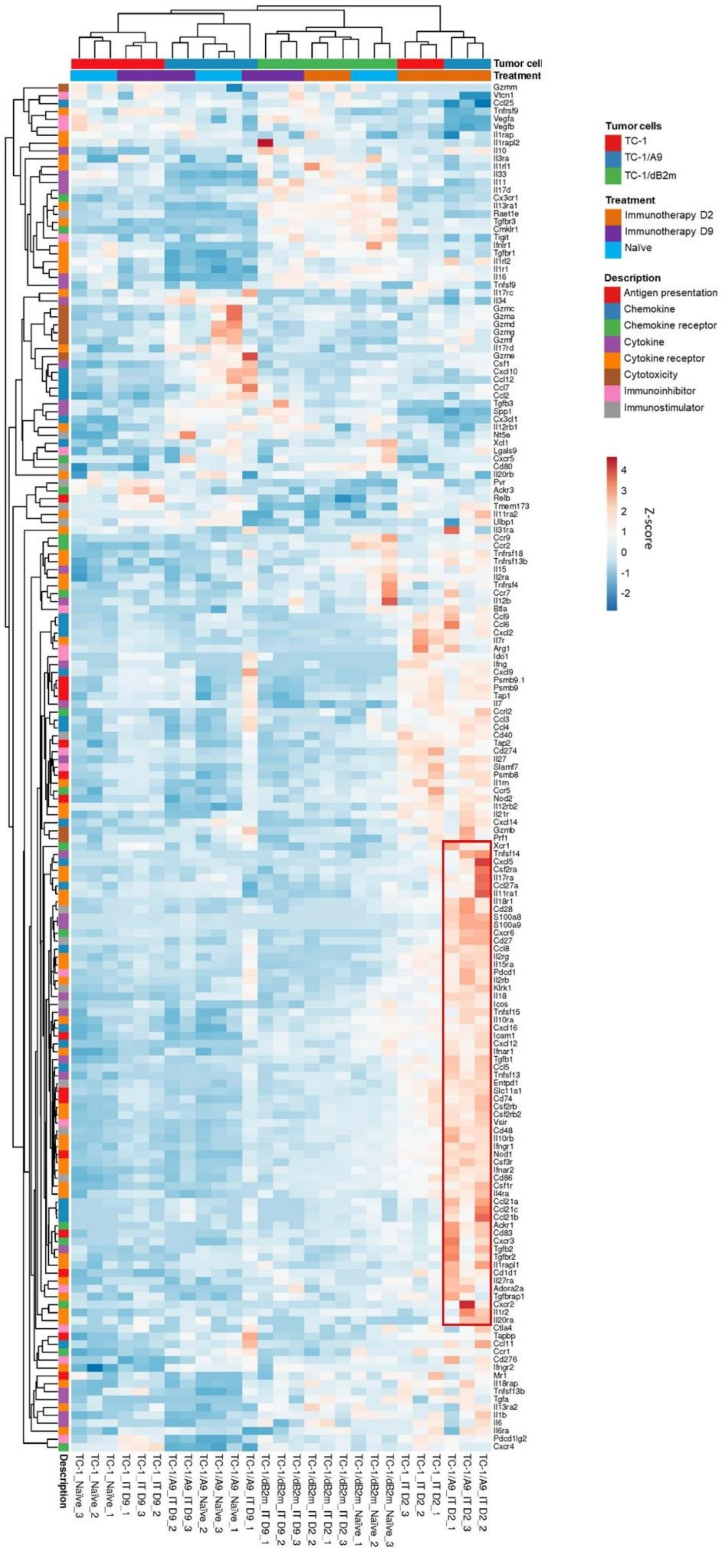

Figure S4. Cluster analysis of immune-related genes. The cluster of genes with higher expression in TC-1/A9- than TC-1-induced tumors 2 days after immunotherapy is indicated.

Table S1. Immune-related genes (*n*=173)

**Antigen presentation**	**Cytokine**	**Cytokine receptor**	**Cytotoxicity**	**Chemokine**	**Chemokine receptor**	**Immunoinhibitor**	**Immunostimulator**
Cd1d1	Csf1	Csf1r	Gzma	Ccl11	Ackr1	Adora2a	Cd27
Cd74	Ifng	Csf2ra	Gzmb	Ccl12	Ackr3	Arg1	Cd28
Cd83	Il10	Csf2rb	Gzmc	Ccl2	Ccr1	Btla	Cd40
Icam1	Il11	Csf2rb2	Gzmd	Ccl21a	Ccr2	Cd274	Cd48
Mr1	Il12b	Csf3r	Gzme	Ccl21b	Ccr5	Cd276	Cd80
Nod1	Il15	Ifnar1	Gzmf	Ccl21c	Ccr7	Ctla4	Cd86
Nod2	Il16	Ifnar2	Gzmg	Ccl25	Ccr9	Ido1	Entpd1
Psmb8	Il17d	Ifngr1	Gzmm	Ccl27a	Ccrl2	Lgals9	Icos
Psmb9	Il18	Ifngr2	Prf1	Ccl3	Cmklr1	Pdcd1	Klrk1
Psmb9	Il1b	Ifnlr1		Ccl4	Cx3cr1	Pdcd1lg2	Nt5e
Relb	Il27	Il10ra		Ccl5	Cxcr2	Slamf7	Pvr
Slc11a1	Il33	Il10rb		Ccl6	Cxcr3	Tigit	Raet1e
Tap1	Il34	Il11ra1		Ccl7	Cxcr4	Vegfa	Tmem173
Tap2	Il6	Il11ra2		Ccl8	Cxcr5	Vegfb	Ulbp1
Tapbp	Il7	Il12rb1		Ccl9	Cxcr6	Vsir	
	Spp1	Il12rb2		Cx3cl1	Xcr1	Vtcn1	
	Tgfa	Il13ra1		Cxcl10			
	Tgfb1	Il13ra2		Cxcl12			
	Tgfb2	Il15ra		Cxcl14			
	Tgfb3	Il17ra		Cxcl16			
	Tnfsf13	Il17rc		Cxcl2			
	Tnfsf13b	Il17rd		Cxcl5			
	Tnfsf14	Il18r1		Cxcl9			
	Tnfsf15	Il18rap		Xcl1			
	Tnfsf9	Il1r1					
	S100a8	Il1r2					
	S100a9	Il1rap					
		Il1rapl1					
		Il1rapl2					
		Il1rl1					
		Il1rl2					
		Il1rn					
		Il20ra					
		Il20rb					
		Il21r					
		Il27ra					
		Il2ra					
		Il2rb					
		Il2rg					
		Il31ra					
		Il3ra					
		Il4ra					
		Il6ra					
		Il7r					
		Tgfbr1					
		Tgfbr2					
		Tgfbr3					
		Tgfbrap1					
		Tnfrsf13b					
		Tnfrsf18					
		Tnfrsf4					
		Tnfrsf9					
